# High-Transconductance, Highly Elastic, Durable and Recyclable All-Polymer Electrochemical Transistors with 3D Micro-Engineered Interfaces

**DOI:** 10.1007/s40820-022-00930-5

**Published:** 2022-09-12

**Authors:** Wenjin Wang, Zhaoxian Li, Mancheng Li, Lvye Fang, Fubin Chen, Songjia Han, Liuyuan Lan, Junxin Chen, Qize Chen, Hongshang Wang, Chuan Liu, Yabin Yang, Wan Yue, Zhuang Xie

**Affiliations:** 1 School of Materials Science and Engineering, Guangzhou Key Laboratory of Flexible Electronic Materials and Wearable Devices and Key Laboratory for Polymeric Composite and Functional Materials of Ministry of Education, Sun Yat-Sen University, Guangzhou, 510275 People’s Republic of China; 2 State Key Laboratory of Optoelectronic Materials and Technologies and Guangdong Province Key Laboratory of Display Material and Technology, School of Electronics and Information Technology, Sun Yat-Sen University, Guangzhou, 510275 People’s Republic of China

**Keywords:** Conducting polymer, Gelatin organohydrogel electrolyte, Organic electrochemical transistor, Stretchable electronics, Soft lithography

## Abstract

Organic electrochemical transistors (OECTs) have emerged as versatile platforms for broad applications spanning from flexible and wearable integrated circuits to biomedical monitoring to neuromorphic computing. A variety of materials and tailored micro/nanostructures have recently been developed to realized stretchable OECTs, however, a solid-state OECT with high elasticity has not been demonstrated to date. Herein, we present a general platform developed for the facile generation of highly elastic all-polymer OECTs with high transconductance (up to 12.7 mS), long-term mechanical and environmental durability, and sustainability. Rapid prototyping of these devices was achieved simply by transfer printing lithium bis(trifluoromethane)sulfonimide doped poly(3,4-ethylenedioxythiophene): poly(styrene sulfonate) (PEDOT:PSS/LiTFSI) microstructures onto a resilient gelatin-based gel electrolyte, in which both depletion-mode and enhancement-mode OECTs were produced using various active channels. Remarkably, the elaborate 3D architectures of the PEDOT:PSS were engineered, and an imprinted 3D-microstructured channel/electrolyte interface combined with wrinkled electrodes provided performance that was retained (> 70%) through biaxial stretching of 100% strain and after 1000 repeated cycles of 80% strain. Furthermore, the anti-drying and degradable gelatin and the self-crosslinked PEDOT:PSS/LiTFSI jointly enabled stability during > 4 months of storage and on-demand disposal and recycling. This work thus represents a straightforward approach towards high-performance stretchable organic electronics for wearable/implantable/neuromorphic/sustainable applications.

**Supplementary Information:**

The online version contains supplementary material available at 10.1007/s40820-022-00930-5.

## Highlights

- Facile fabrication of high-transconductance (>10 mS) and highly elastic all-polymer organic electrochemical transistors was presented using gelatin-based electrolyte supporting printed PEDOT:PSS/LiTFSI microstructures.
- PEDOT:PSS/LiTFSI wrinkled microelectrodes and imprinted 3D-microstructured channel/electrolyte interface allowed biaxial stretchability of 100% strain and performance preservation after 1000 cycles of 80% strain.
- The glycerol-soaked elastic gelatin electrolyte also permitted long-term environmental stability for months and enabled readily recyclable device, paving the way to wide applications spanning from artificial synapses to wearable sensing.

**Supplementary Information:**

The online version contains supplementary material available at 10.1007/s40820-022-00930-5.

## Graphical abstract

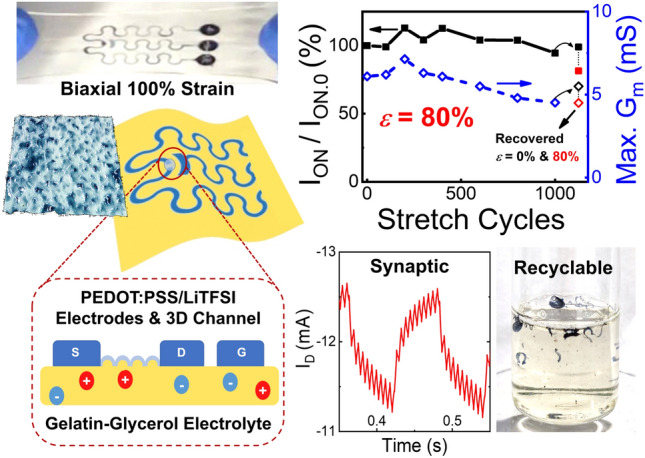

## Introduction

Conducting-polymer-based organic electrochemical transistors (OECTs) are playing increasingly pivotal roles in the booming development of fields including smart tags, wearable electronics, continuous biomonitoring patches and neuromorphic computing devices, with their low operating voltages (< 1 V) and compatibility with aqueous environments providing extensive opportunities for power-saving and bioelectronic devices [1, 2, 3, 4, 5]. Unlike inorganic semiconductor chips, OECTs can be fabricated easily via low-cost and large-area printing techniques with inherent flexibility by utilizing the solution-processable polymer materials, which can meet the growing demand for electronic devices that can be bent, folded, or even stretched [2, 6]. More recently, all-solid-state OECTs incorporated with gel electrolytes have also attracted considerable attentions [7, 8]. These soft gels can be made from biocompatible and biodegradable material that enable conformal and intimate contact with skin or organs to aid signal recording owing to their tissue-like softness [9, 10]. Use of environmentally stable gel electrolytes further prolongs device lifetimes under a wide range of conditions [11, 12] and allows long-term continuous monitoring [10]. Furthermore, gels with mechanically tough networks also function as flexible support structures for electrodes without the need for additional substrates, thus simplifying the processes required for device fabrication, waste recycling or implant bioabsorption [9, 13]. In addition, the gel networks that are capable of encapsulating various ionic or bioactive species also permit the development of customized physio-/chemo-/bio-sensing and neuromorphic platforms [14, 15, 16], which would expand the application scopes of OECTs significantly.

Nevertheless, there are a very limited number of researches in achieving all-solid-state OECTs with high elasticity to date. The lack of the highly stretchable conductors and semiconducting polymers required, along with the elastic gel electrolytes, has greatly hindered the development of mechanically robust OECTs that are capable of sustaining large strains of > 50% [17, 18]. Major efforts have been devoted to developing stretchable poly(3,4-ethylenedioxythiophene):poly(styrene sulfonate) (PEDOT:PSS)-based conducting polymers that exhibit steady electronic functionality under applied strains [19, 20, 21, 22, 23, 24]. A few recent works also explored microstructures that were designed to improve the stretchability of OECTs. For example, wrinkled metal electrodes [10, 25] and metal grid arrays [26] were demonstrated to achieve stable transconductance (> 1 mS) under strains of up to ~ 30%. Microscopically cracked metal electrodes [27] and metal nanowires embedded in elastomers [22] could allow retained device performances with respect to strains of 50%. OECTs based on either in-plane [28] or out-of-plane [29] curved conducting polymer microwires and woven fabric structures [30] showed promisingly high stretchability, but the fabrication processes are complicated. Additionally, Yan’s group reported printing of conducting polymers on biomimicking 3D microtextured templates to realize omnidirectional stretchability for up to 30% strain [31]. Very recently, Chen and colleagues also developed an intrinsically stretchable semiconducting channel material with honeycomb-like microcavities to resist thousands of cycles of biaxial strains at 30% [18]. Nevertheless, for gel-based OECTs, the permanent shape changes and interfacial delamination that may occur during repeated deformation have remained as crucial challenges [13], and the stability of the coupled electronic-ionic conducting interface also requires detailed study over long periods. In addition, the prior research mostly employed high-cost and brittle metal electrodes and rubbery substrates, thus adding unfavorable sustainability issues related to treatment of electronic wastes [32, 33].

Herein, we developed an all-polymer platform and a set of patterning strategies to facilitate the generation of substrate-free and highly elastic OECTs (> 100% strain), which displayed 3D micro-engineered conducting polymer/gel electrolyte interfaces, high transconductance, long-term mechanical and environmental durability and recyclability. This was achieved by exploiting a gelatin-based resilient and degradable organohydrogel electrolyte (gelatin-glycerol/sodium citrate, GEL-GLY/Na_3_Cit) supporting patterned lithium bis(trifluoromethane)sulfonimide (LiTFSI)-doped PEDOT:PSS (PEDOT:PSS/LiTFSI) microstructures, which was capable of serving as both stable soft electrodes and high-performance active channel layer. A straightforward transfer printing approach was developed to enable rapid prototyping of the all-polymer OECTs with diverse electrode configurations, and 3D microscale channel topographies were also fabricated via imprinting using microstructured elastomeric polydimethylsiloxane (PDMS) molds. Furthermore, the mechanoelectrical responses of the gel electrolyte, the conducting polymer films, and the complete OECT device were clarified, with both wrinkled and honeycomb-like 3D-microstructured electrode/electrolyte interfaces being evaluated in terms of their resistance to omnidirectional deformation under tensile strains of > 100%. Then, proof-of-concept on-skin, synapse-mimicking, and biosensing applications of the all-polymer OECTs were demonstrated. Finally, the long-term stability of the electrode/electrolyte interface was studied over a period of more than four months, and the simple disposal and recycling processes of the all-polymer devices were verified. The straightforward approaches presented here will promote the application of a wide range of organic electronic materials to produce wearable and implantable devices with sustainability.

## Experimental Section

### Materials

Gelatin (GEL, ~ 240 g Bloom), glycerol (GLY, ≥ 99.5%), sodium citrate (Na_3_Cit, 98%) bis(trifluoromethane)sulfonimide lithium salt (LiTFSI, 99%), glucose oxidase, and glucose (96%) were purchased from Aladdin Chemical Reagent Co., Ltd. PEDOT:PSS (PH1000, 1.1–1.3 wt.% solid content) was purchased from Heraeus Co., Ltd. Polydimethylsiloxane (PDMS, Sylgard 184) was obtained from Dow Corning. Poly(triethylene glycol cyclopenta[2,1-b:3,4-b′]dithiophene-monoethylene glycol bithiophene) (P3gCPDT-1gT2) was synthesized according to literature [34].

### Preparation and Characterization of Gelatin Organohydrogel Electrolytes

5 g gelatin (25 wt%) was dissolved in 15 mL deionized (DI) water and heated at 60 °C until it turned into a clear solution. Then the solution was cast into a mold and frozen at 4 °C for 1 h to obtain the hydrogel. Organohydrogel electrolytes GEL-GLY/Na_3_Cit were prepared by immersing the as-made hydrogels in the 0.2 M Na_3_Cit solution with 60% v/v glycerol for 3 h at room temperature (26 ± 2 °C) [35, 36]. The tensile measurements of the as-prepared gel electrolytes were carried out using a universal material testing machine (HZ1004B, Dongguan Lixian). For the tensile stress–strain test, a 2 mm wide and 1.5 mm thick dumbbell gel electrolyte sample with a gauge length of 15 mm was used, and the tensile rate was 50 mm min^–1^. For tensile adhesion measurement, the gels were cut into 20 mm × 20 mm samples. Then, the PEDOT:PSS/LiTFSI films on a stainless-steel support were attached with the gel on both sides. The assembled plates were clamped to the HZ1004B and then separated at a shear speed of 10 mm min^–1^. The ion conductivities (mS cm^–1^) and capacitance of the gel electrolytes were measured by electrochemical impedance spectroscopy (EIS) using an electrochemical workstation (CHI 660E, Shanghai Chenhua) in the range of 1–10^5^ Hz. The ion conductivity was calculated by *σ* = *H*/(*R* × *A*), where *H* (cm), *A* (cm^2^), and *R* (Ω) are the gel thickness, electrode area, and bulk resistance obtained from Nyquist plots, respectively.

### Patterning of PEDOT:PSS/LiTFSI on Gel Electrolyte

The surface of PDMS was covered with a shadow mask and treated with oxygen plasma to produce a patterned hydrophilic and hydrophobic surface. PEDOT:PSS added with LiTFSI (0.2 to 2 wt%) was vigorously stirred for at least 1 h and used within 1 day. Then it could be diluted with deionized H_2_O for adjustment of the film thickness. After dropping PEDOT:PSS/LiTFSI solution onto the patterned PDMS, the solution automatically accumulated on the hydrophilic part by dewetting to form PEDOT:PSS/LiTFSI patterns. After baking at 130 °C for 15 min, the polymer patterns were transferred from PDMS onto a piece of GEL-GLY/Na_3_Cit under slight pressure for a few seconds. To prepare wrinkled patterns, the gel was prestretched to certain strains and attached with the polymer thin film, followed by releasing to the relaxed state. To generate 3D-microstructured patterns, PEDOT:PSS/LiTFSI (1:2 dilution) was spin coated (1000–3000 rpm, 60 s) onto a microstructured PDMS mold and annealed, and the hot mold was immediately contacted with GEL-GLY/Na_3_Cit under ~ 10 g weight for 1 min. After cooling to room temperature, the PDMS mold was removed to leave imprinted gel microstructures coated with thin PEDOT:PSS/LiTFSI layer.

### Fabrication and Characterization of OECTs

Au electrodes (10 nm Cr and 100 nm Au) were deposited on glass substrates by thermal evaporation using a shadow mask. The channel area between the source and drain was 5 mm × 0.2 mm. 5 μL of PEDOT:PSS/LiTFSI solution was drop cast on both channel and gate regions that were treated with oxygen plasma, followed by removing the thicker coffee rings at the edge if existing, in which the coating on the gate electrode was maintained at the same thickness using undiluted PEDOT:PSS/2% LiTFSI. Then GEL-GLY/Na_3_Cit was placed on the top to cover the channel and gate electrodes. All-polymer OECTs were prepared by transferring both polymer electrodes and channel layer onto the gel surface, in which the channel materials could be deposited between the electrode gaps on PDMS and transferred in one step, or the electrode and channel layers could be printed separately. Output and transfer characterizations of OECTs were performed with a Keithley semiconductor parametric analyzer (model 2612B). In output curve measurements, the drain voltage (*V*_D_) was swept from 0.5 to − 1 V while the gate voltage (*V*_G_) was altered with steps of 0.2 or 0.4 V. In transfer curve measurements, the *V*_D_ was maintained as − 0.5 V and the sweep speed of *V*_G_ was set as 0.5 mV s^−1^. The transconductance was calculated from the transfer curve with the formula:

1 $$G_{m} = \frac{{\partial I_{{\text{D}}} }}{{\partial V_{{\text{G}}} }}.$$

Synapse-mimicking performances were measured using a semiconductor characterization system (PDA FS Pro).

## Results and Discussion

### Performances of GEL-GLY/Na_3_Cit and PEDOT:PSS/LiTFSI

**Fig. 1:**
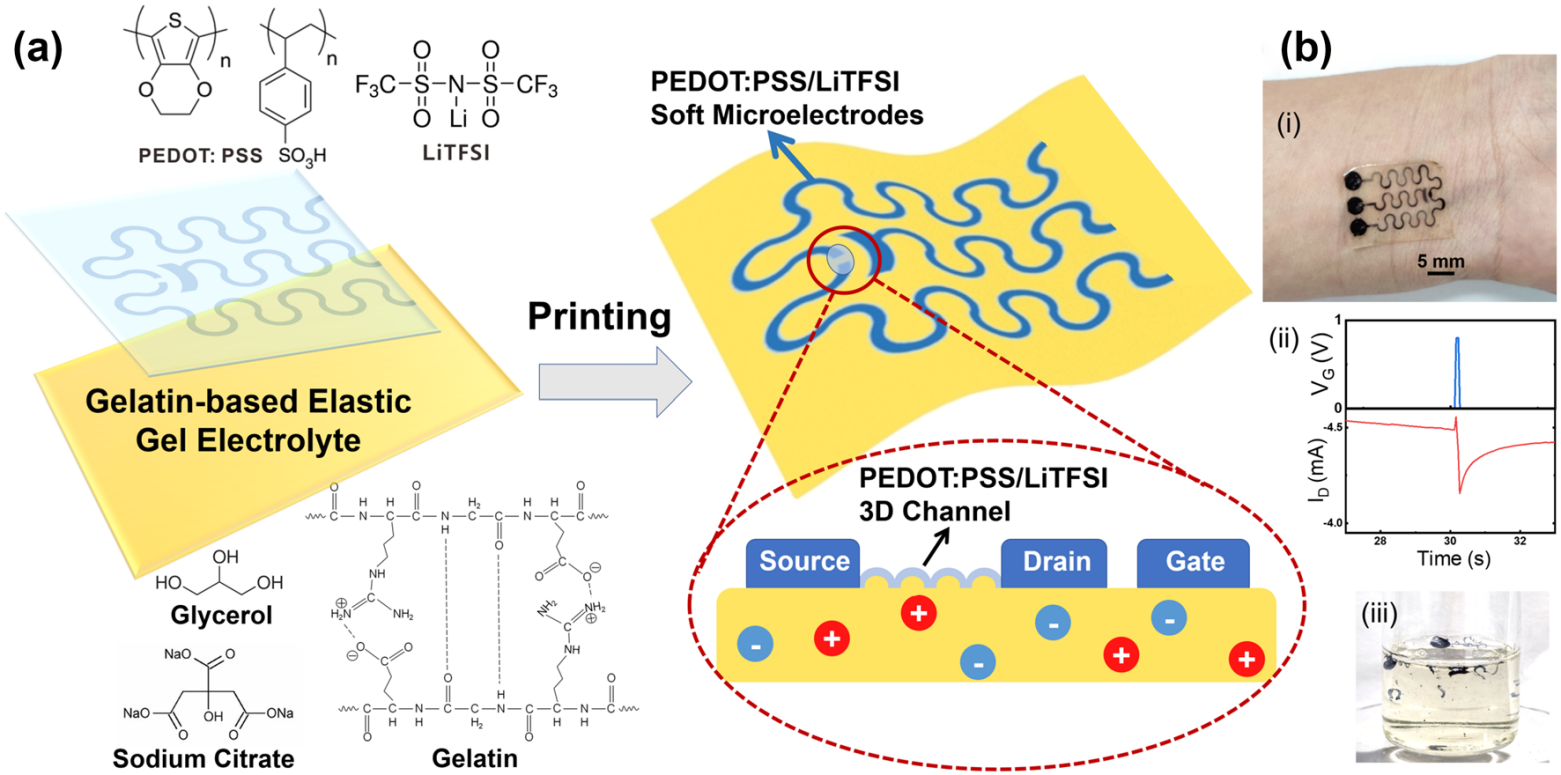
**a** Schematic diagram of substrate-free all-polymer organic electrochemical transistor (OECT) utilizing gelatin-glycerol/sodium citrate (GEL-GLY/Na_3_Cit) organohydrogel electrolyte as the elastic substrate, and PEDOT:PSS/LiTFSI as both the soft electrodes and the active channel layer with 3D micro-engineered interface. **b** Images show that the printed all-polymer OECT can be body-attachable (i), synapse-mimicking with slower current recovery after a voltage spike (ii), and readily recyclable with water (iii)

The all-polymer OECT device can be constructed simply on a tough gel-based solid-state electrolyte that is attached with PEDOT:PSS-based source, drain and gate electrodes, along with the active channel layer, as shown in Fig. 1a. The electrochemical doping/de-doping process at the channel interface accompanied with ion flow through the solid-state electrolyte enables the modulation of the channel conductance, leading to a source-drain current (*I*_D_) that is switchable via the applied gate voltage (*V*_G_). The utilization of ion-conducting gels or elastomers combined with the PEDOT:PSS electrodes have been reported previously to realize substrate-free all-organic field-effect transistors [9, 13, 37, 38], but the high-performance and stretchable all-polymer OECTs have yet to be demonstrated to date. To this end, a multifunctional elastic organohydrogel electrolyte GEL-GLY/Na_3_Cit was first developed consisting of gelatin (GEL) from natural sources, glycerol (GLY) as an anti-drying agent and sodium citrate (Na_3_Cit) to act as the electrolyte. The resulting gel material could possess tunable toughness, good resilience, universal adhesiveness, wide environmental adaptivity and recyclability at the mean time [36, 39]. It is worth pointing out that, since the electrodes are constantly in contact with the conductive electrolyte, the *I*_D_ recorded in the all-polymer OECT may include both the electronic current in the channel layer and the ionic current from the underlying electrolyte. The background ionic current may then determine the lowest current that occurs in the OFF state and reduce the ON/OFF ratio as well. Therefore, the conductivity contrast between the channel material and the gel electrolyte should be as high as possible. Consequently, high-content glycerol (60–80%) could be incorporated in the gel electrolyte to lower the ion conductivity and minimize the ionic current [40]. Next, PEDOT:PSS doped with LiTFSI was selected as the high-conductivity and stretchable electronic conductors. LiTFSI as low as 1 wt% was reported to promote internal phase separation and the formation of long-range ordered conductive networks, and it could also act as a “secondary” dopant for PEDOT [19, 41]. Besides, LiTFSI can induce interchain crosslinking of PSS as well. Therefore, LiTFSI doping leads to boosted conductivity of up to > 2000 S cm^−1^ with simultaneously improved stretchability ranging from 20 to > 100% strain depending on the film thickness and dopant ratio [19, 41, 42, 43, 44]. Furthermore, the transconductance (*G*_*m*_) of OECT in the saturation regime can be defined as follows:

2 $$G_{m} = \frac{Wd}{L}\mu C^{*} \left( {V_{{\text{T}}} - V_{{\text{G}}} } \right)$$

where *W*, *L*, and *d* are the channel width, length and thickness, respectively, *μ* is the carrier mobility, C^*^ is the volume capacitance, and *V*_*T*_ is the threshold voltage [45]. As a result, LiTFSI doping may contribute to a high *G*_*m*_ because of the improved μC* figure of merit [46]. Therefore, PEDOT:PSS/LiTFSI is expected to serve as a superior soft alternative to metal electrodes, and a high-transconductance channel. The combination between the gelatin organohydrogel electrolyte and PEDOT:PSS/LiTFSI would enable stretchable all-polymer OECTs with diverse functionalities (Fig. 1b).

**Fig. 2:**
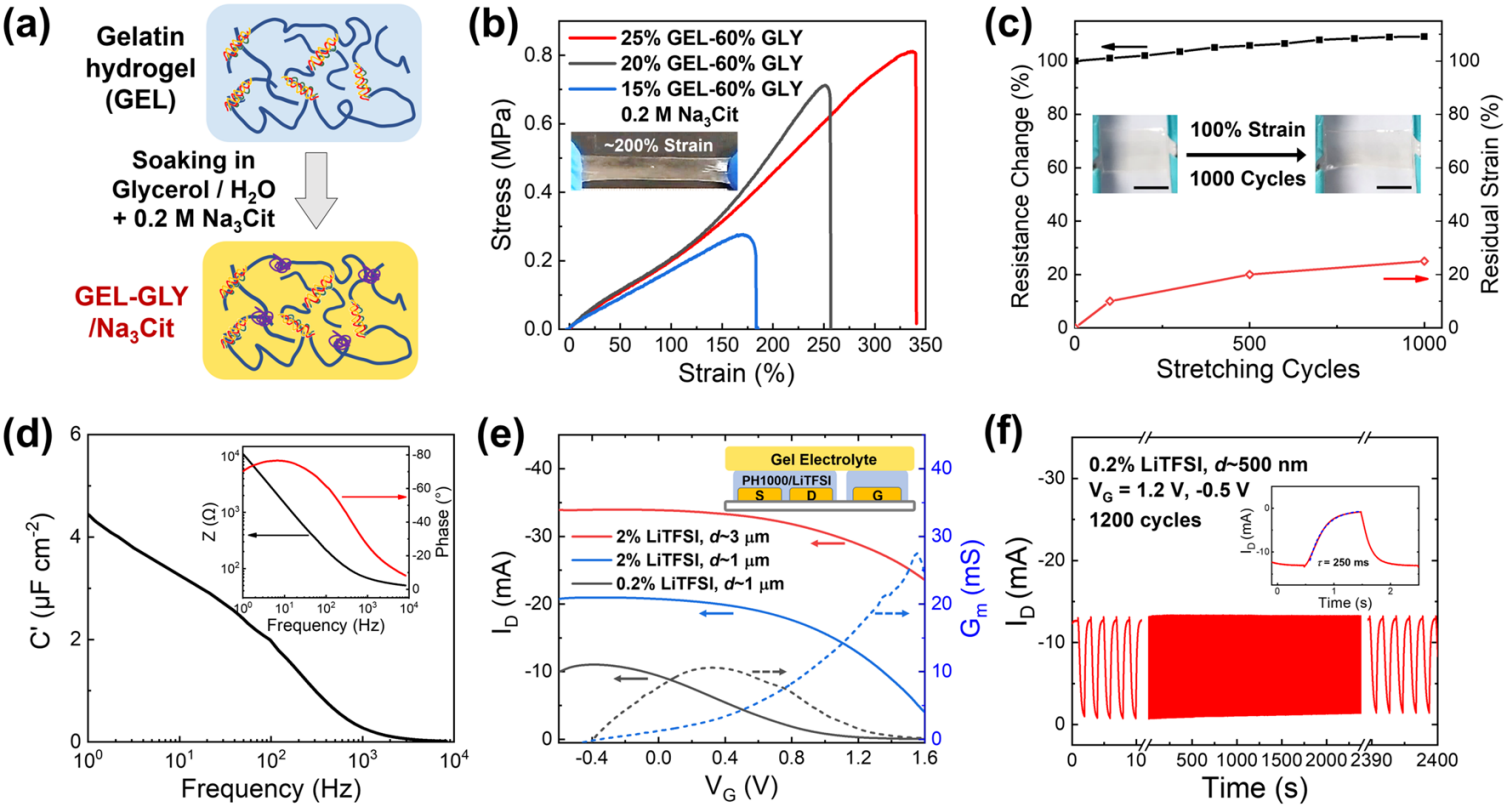
**a** Preparation process of gelatin electrolyte by solvent exchange with glycerol solution containing 0.2 M sodium citrate. **b** Tensile stress–strain curves of the as-prepared GEL-GLY/Na_3_Cit having various gelatin compositions. Inset photo shows the gel electrolyte withstanding ~ 200% strain. **c** The percentages of resistance change and residual elongation of 25% GEL–60% GLY/Na_3_Cit during 1000 cycles of stretching at 100% strain. Inset photos indicate the slight shape change after the stretching cycles, scale bars are 1 cm. **d** Frequency-dependent specific capacitance of the gel electrolyte at 1 to 10^4^ Hz. Inset shows the plot of phase angle and impedance with frequency. **e** Representative transfer curves with the corresponding transconductance (*G*_*m*_) progressions of planar OECTs composed of GEL-GLY/Na_3_Cit solid-state electrolyte and LiTFSI-doped PEDOT:PSS (PH1000) channel materials on Au electrodes (*W/L* = 5000/200 μm). The conductance modulation behavior was tuned by varying the channel film thickness or LiTFSI concentration. All the gate electrodes were coated with PEDOT:PSS/2% LiTFSI (*d* ≈ 3 μm). **f** Plot of long-term *I*_D_ switching with alternating *V*_G_ between − 0.5 and 1.2 V with a 1 s pulse period. Inset displays the response time (*τ*) of ~ 250 ms upon applying the *V*_G_ of 1.2 V, as estimated by exponential fitting. The channels were biased at *V*_D_ =  − 0.5 V in (**e**) and (**f**)

To realize this elastic gelatin electrolyte, a soaking strategy was applied to toughen the fragile gelatin hydrogel via solvent exchange with a glycerol solution (60% v/v) containing 0.2 M Na_3_Cit (Fig. 2a) [35, 36], and this process yielded the organohydrogel electrolyte GEL-GLY/Na_3_Cit with remarkably enhanced mechanical properties. As shown in Fig. 2b, the gelatin organohydrogel became rubbery after soaking for 3 h and could then sustain a tensile strain of ~ 200% in a reversible manner without fracture. This improved toughness could be a result of the increased interactions among the protein chains and the formation of multiple hydrogen bonds between the glycerol and gelatin networks after replacement of water with glycerol, as well as possible salting-out effect of Na_3_Cit [35, 36]. The tensile stress–strain characterizations in Fig. 2b indicated that the stretchability and toughness could be adjusted by varying the gelatin content, while the elastic modulus was maintained at ~ 200 kPa. For example, an increase in the gelatin composition from 15 to 25 wt% led to improved stretchability from 180 to 340% strain because of the formation of higher numbers of triple-helix joints and greater chain entanglement. Furthermore, in the presence of Na_3_Cit, the gel functioned as an ionic conductor and showed stable resistance after repeated stretching. As Fig. 2c shows, after 1000 stretching cycles at 100% strain, the gel conductor (25% GEL–60% GLY/0.2 M Na_3_Cit) showed a < 10% increase in resistance parallel to the stretching direction. This slight change may be a consequence of the suppressed irreversible elongation after cyclic stretching (< 25% residual strain) by taking advantage of the self-recovery characteristics of the noncovalently crosslinked gel networks (Fig. S1) [47]. Impedance measurement of GEL-GLY/Na_3_Cit was further performed to determine both the internal ion conductivity and the capacitance of the solid-state electrolyte (Fig. 2d). Average ion conductivity of 1.5 ± 0.2 mS cm^−1^ was calculated for the above gel electrolytes as described in the experimental section. And a maximum capacitance of 4.9 µF cm^−1^ was determined at 1 Hz. The phase angle could approach − 70° to − 80° below 100 Hz, which was close to that of an ideal capacitor (− 90°). Such excellent mechanical resilience and electrical properties of GEL-GLY/Na_3_Cit thus established it as a suitable basis for highly elastic OECTs without the need for additional rubbery supports.

Next, in order to evaluate the performance of PEDOT:PSS/LiTFSI for use in an all-solid-state gel-based OECT, we began by fabricating devices using Au electrodes. The high-conductivity PEDOT:PSS aqueous solution (PH1000, 1.1–1.3 wt% solid content) mixed with LiTFSI was drop cast onto two pairs of Au electrodes as channel and gate, respectively, where the film thickness in the channel region could be adjusted down to ~ 100 nm via dilution (Fig. S2a), while the coating on the gate electrode remained the same (~ 3 µm) using undiluted PEDOT:PSS/2% LiTFSI. After addition of 2 wt% LiTFSI, the as-prepared PEDOT:PSS/LiTFSI films demonstrated conductivities of up to ~ 2000 S cm^−1^ for thin films of < 200-nm thick and > 300 S cm^−1^ for thicker films on the scale of a few microns, which was consistent with previous reports [41, 43]. Then, a piece of GEL-GLY/Na_3_Cit was placed on top of the conducting polymer films such that it bridged the channel and gate regions. When a positive *V*_G_ was applied, the positively charged sodium ions (Na^+^) in the gel electrolyte were drifted and injected into the active layer, resulting in compensation of PSS and the reduction of conducting PEDOT^+^ to PEDOT^0^, that is, the insulating neutral state [12]. We investigated the conductance modulation of PEDOT:PSS/LiTFSI by applying *V*_G_ over a range from − 0.6 to 1.6 V while maintaining a constant drain voltage (*V*_D_) of − 0.5 V. Efficient gating in the OECT requires the gate electrode to have a high capacitance that should be > 10 times higher than the channel capacitance to avoid the drop of the applied *V*_G_ at the gate-electrolyte interface [1]. Accordingly, when thick films were deposited on both the channel and the gate by applying undiluted PEDOT:PSS/2% LiTFSI solution, the *I*_D_ with a channel thickness of ~ 3 µm could initially surpass 34 mA or even 40 mA, and then only decreased slightly over the *V*_G_ range up to 1.6 V, leading to > 70% preservation of the conductance (Fig. 2e). This indicated that the sufficiently thick film with high LiTFSI ratio could be hardly de-doped over a wide electrochemical window because of the reduction in the effective *V*_G_ at the channel-electrolyte interface and the hindered penetration of ions into the thick conducting polymer layer. Therefore, the thick PEDOT:PSS/2% LiTFSI films showing stable electronic behavior under applied *V*_G_ could be suitable to serve as soft polymer electrodes in the all-polymer OECT.

In fact, by lowering the channel capacitance through reducing the film thickness, we were also able to tune the transfer characteristics of PEDOT:PSS/LiTFSI to attain the required high-performance OECTs as well. For instance, thinner channels with thickness of ~ 1 µm that were deposited from 1:8 diluted PEDOT:PSS/LiTFSI solutions showed a more distinct drop in *I*_D_ with increasing *V*_G_, corresponding with a typical feature of depletion-mode OECT on the basis of de-doping of p-type PEDOT:PSS at the reduction potential. Typically, with 2 wt% LiTFSI, the *I*_D_ remained almost stable at *V*_G_ < 0.6 V with an *I*_ON_ of 21 mA, and it decreased to 1/5 of its original value at *V*_G_ = 1.6 V (Figs. 2e and S2b). The maximum *G*_*m*_ of 28 mS appeared at *V*_G_ ≈ 1.5 V, and *V*_T_ was estimated to be ~ 1.9 V. Following further reduction of the LiTFSI content to 0.2 wt%, the OECT was capable of operation at even lower voltages (Figs. 2e and S2c) with a higher ON/OFF ratio (> 100), in which the values of *V*_G_ at the peak *G*_*m*_ and *V*_T_ were shifted to ~ 0.3 and ~ 1.4 V, respectively, despite the reduction in both *I*_ON_ (11 mA) and the maximum *G*_*m*_ value (10.7 mS). Tuning of the film thickness of the PEDOT:PSS/0.2% LiTFSI channel could also be used to adjust the *G*_*m*_ (Fig. S2d–e), and a high *G*_*m*_ of 35 mS at *V*_G_ = 1 V and an ON/OFF ratio of > 500 were achieved by using a 2.7-µm-thick channel. In addition, the PEDOT:PSS/0.2% LiTFSI channel (*d* ≈ 500 nm) was electrochemically stable and exhibited almost unchanged *I*_D_ oscillations (> 95% retention) when pulsed *V*_G_ was applied between − 0.5 and 1.2 V for 1200 cycles (Fig. 2f). During the cycling of the gel-based OECT, the positive *V*_G_ of 1.2 V caused the switch of *I*_D_ from ~ 13 to ~ 0.8 mA with a response time of ~ 250 ms (Fig. 2f inset). This longer response time in comparison with OECTs under liquid electrolyte (< 1 ms) [15] could be mainly ascribed to the lower ion mobility in the organohydrogel electrolyte as well as the large channel volume, since the response time is proportional to the electrolyte resistance and the channel capacitance [1]. We also measured the transient response of PEDOT:PSS/LiTFSI channels in 0.1 M NaCl aqueous electrolyte with reduced channel dimensions (*W/L* = 1000/200 µm, *d* ≈ 100 nm, Fig. S3). The lowest response time could reach ~ 9.8 ms for the channel added with 0.2 wt% LiTFSI, while the channel with 2 wt% LiTFSI showed slower response (~ 18.2 ms). This indicated that LiTFSI dopant might also affect the device’s transient response.

Based on the tunable transfer characteristics of PEDOT:PSS/LiTFSI in the presence of a solid-state gelatin electrolyte, fabrication of high-transconductance and reliable all-polymer OECTs on the supporting gel electrolyte could be made feasible by addressing the following two issues. First, the source, drain and gate electrodes could utilize the thick PEDOT:PSS/2% LiTFSI films with thickness of at least 3 µm to assure low resistances. Also, the capacitance ratio between source/drain and gate electrodes should be high enough to ensure that the conductance of the electrodes would not be changed much by the ion motion in the gel electrolyte under the applied *V*_G_. Second, the PEDOT:PSS/LiTFSI channel should be regulated to provide a channel current that is at least one order of magnitude higher than the ionic current (tens of microamperes level), which can be realized by adjusting the channel thickness and the LiTFSI content. Meanwhile, the channel capacitance should be maintained relatively low (< 1/10) compared with the gate electrode, e.g., by reducing the channel area. Efficient gating with a high ON/OFF ratio could thus be enabled in the gel-based all-polymer OECT when the electrode and channel dimensions are optimized.

### Printing and Characterization of All-Polymer OECTs

**Fig. 3:**
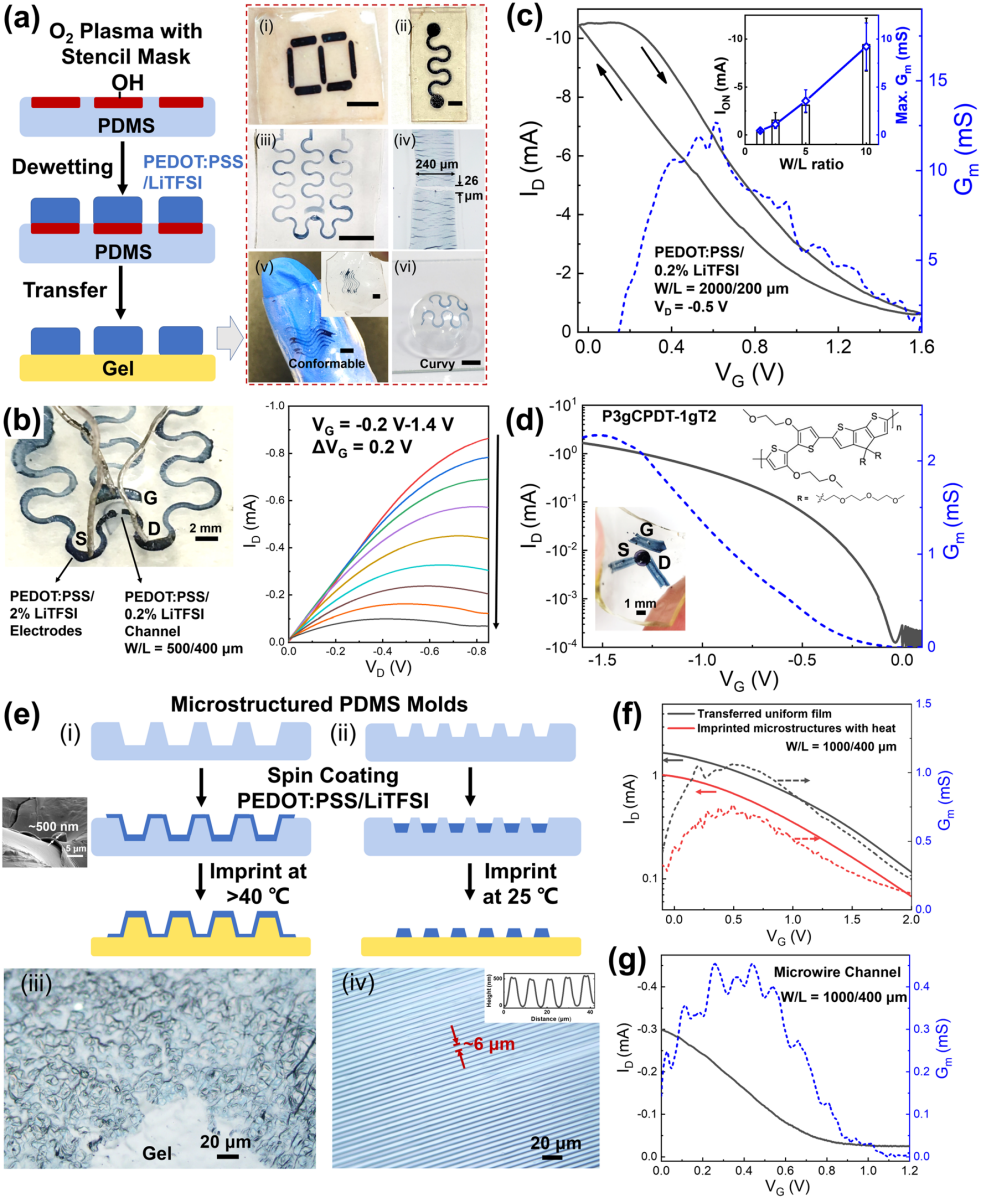
**a** Schematic illustration of the patterning of PEDOT:PSS/LiTFSI by selective dewetting followed by transfer printing of these patterns onto GEL-GLY/Na_3_Cit, including (i) “8”-shape pattern, (ii) serpentine pattern, (iii) stretchable serpentine arrays, (iv) ~ 240-μm-wide line with ~ 26 μm gap formed by cracking, (v) patterns on ultrathin gel film that was conformable to a finger, (vi) patterns printed on contact lens surface with an ultrathin gel coating. Scale bars in all photos are 5 mm. **b** Photo of a typical all-polymer OECT with *W/L* = 500/400 μm and its corresponding output characteristics, in which submicron-thick PEDOT:PSS/0.2% LiTFSI channel layer was printed on top of thick PEDOT:PSS/2% LiTFSI microelectrodes. **c** Typical transfer and *G*_*m*_ curves of the all-polymer OECT with *W/L* = 2000/200 μm. Inset shows the plots of *I*_ON_ and the maximum *G*_*m*_ with *W/L* ratio (*n* = 4). **d** Transfer and *G*_*m*_ curves of an enhancement-mode all-polymer OECT using P3gCPDT-1gT2 as the channel. **e** Schemes (i, ii) and optical microscope images (iii, iv) of PEDOT:PSS/LiTFSI with 3D-microstructured (i, iii) and microwire (ii, iv) morphologies on the GEL-GLY/Na_3_Cit surface by imprinting using PDMS molds. The inserted scanning electron microscopy (SEM) image in (i) and cross-sectional atomic force microscopy (AFM) topography image in (iv) indicated the film thickness of ~ 500 nm in both microstructures. **f** Transfer and *G*_*m*_ curves of OECTs based on imprinted 3D-microstructured and transferred uniform channel layers on the same gel electrolyte. **g** Typical OECT performance of the printed PEDOT:PSS/LiTFSI microwires on gel as the active channel

Next, a straightforward transfer printing strategy was developed to pattern the conducting polymer layers on the gel electrolytes to enable rapid prototyping of the all-polymer OECTs. Dewetting of the PEDOT:PSS/LiTFSI solution was first conducted on a PDMS substrate that had predefined hydrophobic/hydrophilic regions (Figs. 3a and S4a, b). This allowed the polymer microelectrodes to be generated in diverse configurations with linewidths as small as ~ 240 μm (Fig. 3a(i–vi)). The varied pattern appearance from light blue to black also demonstrated that the film thickness could be controlled from submicrometer to above 10 μm in different patterns. In principle, fabrication of polymer electrodes with sub-100-μm width may be possible if superhydrophobic patterns or other high-resolution printing techniques were used [6, 48]. Notably, electrode gaps could also be generated easily by harnessing the crack formation upon bending of the underlying PDMS support, followed by slight stretching of PDMS to tune the gap distance, with the potential to realize gaps as small as ~ 26 μm through carefully tuning the tensile strain (Fig. 3a(iv)). Then, by bringing the PEDOT:PSS/LiTFSI micropatterns into contact with the GEL-GLY/Na_3_Cit surface under a slight pressure, the electrodes could be transferred seamlessly onto the gel electrolyte without leaving residues or damage, as shown in the photographs in Fig. 3a. We found that the gelatin-based gel presented robust adhesiveness with PEDOT:PSS that allowed the polymer thin films to peel off easily from PDMS or glass. This behavior could be ascribed to the rich hydrogen-bonding and the electrostatic interactions provided by the protein and PSS chains present at the interface [49]. The adhesion strength between the electrode and the gel electrolyte was measured to be at least 70 kPa, with the PEDOT:PSS film actually breaking before it could be detached from the gel surface. Therefore, the sticky gelatin surface not only permitted easy transfer of the electrodes but also could provide intimate interfacial contact between the active channel layer and the solid-state electrolyte for efficient ion transport. In addition, the PEDOT:PSS/LiTFSI crosslinked rapidly into an insoluble and free-standing stretchable film after drying without addition of other crosslinkers (Fig. S4b inset), which was also beneficial for the high-fidelity pattern transfer. Because of their robust adhesion and intrinsic stretchability, the transferred polymer microelectrodes were highly flexible and were compatible with both flat and curved surfaces. For example, a thin GEL-GLY/Na_3_Cit film with serpentine printed microelectrodes was shown to wrap around a human finger without difficulty (Fig. 3a(v)). In addition, the polymer microelectrodes were printed successfully onto the curved surface of a contact lens that was pre-modified using an ultrathin GEL-GLY/Na_3_Cit coating (Fig. 3a(vi)), indicating their potential for applications in wearable devices and bioelectronics. Additionally, we observed no significant increase in the resistance of PEDOT:PSS/LiTFSI film when it was transferred from PDMS onto the gel surface (Fig. S4c), thus outstanding OECT performance would be allowed.

After the PEDOT:PSS/2% LiTFSI layers were patterned with customized channel *W*/*L* ratios while also retaining sufficient thickness (> 3 μm) to act as electrodes, another thin PEDOT:PSS/LiTFSI layer could be formed in the channel gap to serve as the active channel, thus completing the assembly of the all-polymer OECT. Figure 3b shows a photograph of one as-prepared all-polymer OECT and its output characteristics, in which the semi-transparent thin channel layer (PEDOT:PSS/0.2% LiTFSI, with *d* < 1 μm) was pasted on top of the thick source-drain electrodes showing a dark blue appearance. The electrode width was patterned down to ~ 500 μm and a resistance of < 200 Ω cm^−1^ was attained. Under the condition that *W*/*L* = 500/400 μm, excellent output performance was demonstrated, allowing the reduction of *I*_D_ in the range from ~ 0.8 to ~ 0.08 mA under low-voltage modulation conditions (*V*_G_ < 1.5 V). The maximum *G*_*m*_ was measured as ~ 0.4 mS (Fig. S5a). Furthermore, when the *W*/*L* ratio was increased to 2000/200 μm via the cracking approach, the best all-polymer device exhibited the maximum *G*_*m*_ reaching 12.7 mS at *V*_G_ = 0.6 V, accompanied with a high *I*_ON_ of ~ 10.5 mA and an ON/OFF ratio of ~ 17, as shown in the transfer curve in Fig. 3c. The geometry-normalized transconductance was estimated to be ~ 25.4 S cm^−1^ by measuring the channel thickness as ~ 500 nm. In average both the *I*_ON_ and the maximum *G*_*m*_ were found to be proportional to the *W/L* ratio with good reproducibility (Fig. 3c inset and Fig. S5a, b), in which the batch-to-batch performance could vary with the channel thickness, the dimensional discrepancy of electrodes and the ion conductivity of the gel electrolyte. This geometry-dependent transconductance implied that the OECT performance was largely determined by the de-doping of the thin channel region and that it was less strongly affected by the electrode parts, the conductance of which remained relatively insensitive to *V*_G_. Therefore, control of the gap between thick electrodes could well define the channel region and allow a tunable *G*_*m*_ to be obtained. When compared with the values from previous reports, the *I*_ON_ and *G*_*m*_ values reported here were at least 20-fold higher than those of all-PEDOT OECTs [50] and were comparable or even superior to those of solid-state OECTs with metal electrodes [10, 15, 51], which can be attributed to the high-conductivity PEDOT:PSS/LiTFSI and the geometry of the device design. Additionally, thin channels (*d* < 1 μm) of PEDOT:PSS/2% LiTFSI and other high-performance PEDOT:PSS channel materials such as those doped with ethylene glycol [12] or Triton X-100 [52] have been used to achieve all-polymer OECTs with similarly high transconductance (4–12 mS, see Fig. S5c–e). Rather than limit our work to PEDOT:PSS-based materials, we also attached a thin poly(triethylene glycol cyclopenta[2,1-b:3,4-b′]dithiophene-monoethylene glycol bithiophene) (P3gCPDT-1gT2) [34] layer to the channel region and demonstrated operation of an enhancement-mode all-polymer OECT with high flexibility (Figs. 3d and S5f). As shown in the transfer curve in Fig. 3d, the p-channel OECT showed an *I*_D_ of as low as 10^−4^ mA at *V*_G_ > 0 V, although this current increased rapidly at *V*_G_ < 0 V with a *V*_T_ of − 0.1 V to switch it on. Notably, an impressive ON/OFF ratio that approached ~ 10^4^ and a maximum *G*_*m*_ of 2.3 mS were realized. These values were again comparable to those of a prior metal-electrode-based device [34]. Therefore, this gelatin-based electrolyte with patterned PEDOT:PSS/LiTFSI microelectrodes could provide a versatile soft platform for high-transconductance OECTs that would be compatible with a broad range of active channel materials.

In addition to patterned polymer electrodes, we also used PDMS molds with topological microstructures to engineer the active channel layer, with the aim of realizing 3D microarchitectures. Two types of PDMS mold were used to perform proof-of-concept experiments (Fig. 3e(i and ii)). The first structure was replicated from a sandpaper template to form random microstructures of hollows and protrusions with dimensions on the scale of tens of microns (Fig. S6), which could be exploited to improve the mechanical stretchability of channel layers [31, 53, 54]. The second was formed from lithographically generated microline templates with spacing and height dimensions of ~ 6 and ~ 1 μm, respectively, demonstrating that the pattern dimensions could be pushed down to the sub-10-μm scale. By controlling the spin coating of PEDOT:PSS/LiTFSI onto the plasma-treated PDMS molds, we were able to generate conformally coated thin polymer layers on the coarse microtextures and polymer layers that were confined inside the line grooves [55], in which the film thickness of both microstructures was around 500 nm (Fig. 3e, inserts in i and iv). The mold with random 3D microstructures and PEDOT:PSS/LiTFSI coating was then pressed hard on the GEL-GLY/Na_3_Cit for 1 min while a mold temperature of > 40 °C was maintained to soften the gel surface. After the PDMS mold was separated, the microstructures were imprinted on the gel to form honeycomb-like random bumps coated with PEDOT:PSS/LiTFSI thin layer (Fig. 3e(iii)). To examine whether the imprinted channel/electrolyte interface under heat influence the OECT performance, PEDOT:PSS/0.2% LiTFSI was spin-coated on a PDMS mold including both smooth and 3D-microstructured regions. Then, the uniform thin channel was transfer printed at room temperature from the smooth region, meanwhile the microstructured channel was thermally imprinted onto the same gel electrolyte. The transfer characteristics of the two channels were compared in Fig. 3f, in which the transferred and imprinted channels showed almost identical behavior with slight variations in their *I*_ON_ and *G*_*m*_ values. Therefore, thermal imprinting of the gel electrolyte may not significantly affect the OECT characteristics. In addition, we also fabricated PEDOT:PSS/LiTFSI microwire arrays onto the gel electrolyte by room-temperature imprinting (Figs. 3e(ii, iv) and S7), and then evaluated the OECT performance of the microscale channels through printing of pre-defined polymer microelectrodes to overlap with the microwires (Fig. S7b). These microwire channels, which had *W*/*L* = 1000/400 μm and thicknesses of ~ 500 nm, showed an *I*_ON_ of ~ 0.3 mA and a maximum *G*_*m*_ of 0.45 mS at *V*_G_ < 0.5 V, similar to the values for the uniform channels with *W*/*L* = 500/400 μm. This was reasonable because around half of the area in the microwire arrays was vacant, leading to the reduction of effective *W*. A relatively low *V*_*T*_ of ~ 1 V was also observed, possibly due to the very low channel current that could rapidly decrease to the OFF state at lower *V*_G_. Overall, the above results demonstrated the potential of our all-polymer platform to provide tailored microscale OECT interfaces via simple soft lithographic approaches.

### Stretchability of the All-Polymer OECTs and Proof-of-Concept Applications

**Fig. 4:**
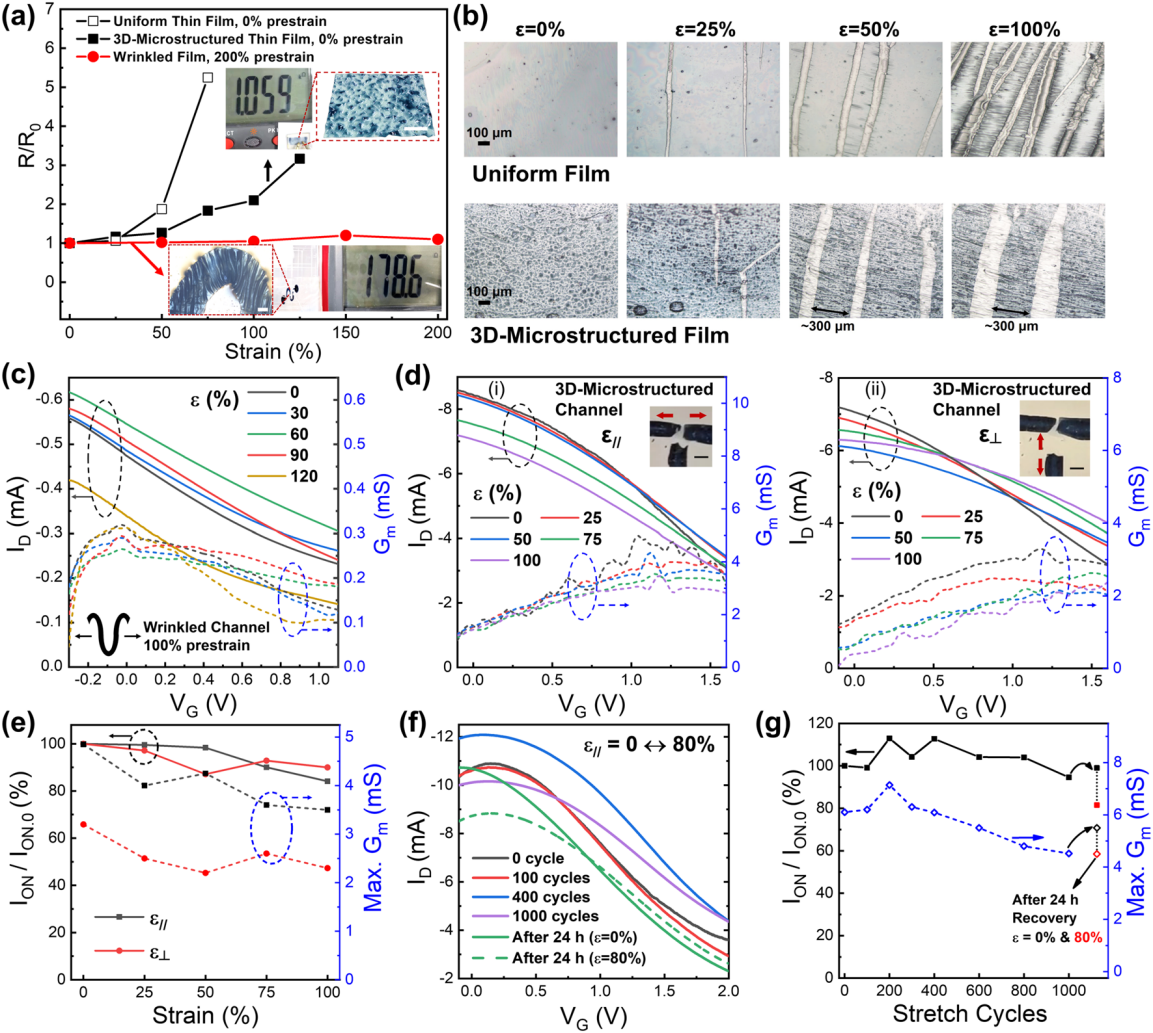
**a** Plot of resistance variation (*R*/*R*_0_) for PEDOT:PSS/LiTFSI films exhibiting three types of morphologies with various strains, including spin-coated uniform and imprinted 3D-microstructured films without prestrain, and wrinkled film with 200% prestrain. Insets display the resistances and microscope images of films with imprinted microstructures and wrinkles, respectively. Scale bars in insets are 200 μm. **b** Optical microscope images of the uniform and 3D-microstructured PEDOT:PSS/LiTFSI films under strains from 0 to 100%. **c** Transfer and *G*_*m*_ curves of the all-polymer OECT with wrinkled channel (100% uniaxial prestrain) under strain from 0 to 120%. **d** Transfer and *G*_*m*_ curves of the all-polymer OECT with 3D-microstructured channel under 0–100% strain in both the parallel (*ε*_//_, i) and perpendicular (*ε*_┴_, ii) directions. Inset photos show the device under ~ 50% strain in each axis, respectively. Scale bars in insets are 1 mm. **e** Plots of *I*_ON_/*I*_ON.0_ and *G*_*m*_ with the strain in the biaxial directions. **f** Transfer curves and **g** plots of *I*_ON_ and *G*_*m*_ variations for the 3D-microstructured channel during the repeated stretching at 80% strain for 1000 cycles. After recovery for 24 h, the device exhibited improved performance and remained highly stretchable to 80% strain. All PEDOT:PSS were added with 2 wt% LiTFSI

The main focus of this study is the realization of highly elastic OECTs, in which the conducting polymer electrodes and active channels limit the mechanical stability. To guarantee a high stretchability, PEDOT:PSS/2% LiTFSI was used for both electrodes and channels. Although the LiTFSI-doped PEDOT:PSS can become an elastic soft film, when the uniform film was attached to the GEL-GLY/Na_3_Cit, cracks continued to appear at strains > 40% (Fig. S8). To the best, ~ 50% of their conductance could be retained at 50% strain for uniform thin films (Fig. 4a). By transferring the polymer films onto pre-stretched gel substrates to form surface wrinkles, the mechanical stretchability could be greatly improved. Because of the soft nature of PEDOT:PSS/LiTFSI, even ultra-thick (> 10 μm) serpentine microelectrodes attached to the gel substrate could form buckled structures upon release while still maintaining resistances of less than 200 Ω (Fig. 4a inset). Furthermore, these wrinkled electrodes with 200% pre-strain could be stretched reversibly within their pre-strain range with few cracks, accompanied by a < 30% resistance variation. As an alternative, the sandpaper-templated 3D-microstructured thin films on the gel electrolyte showed remarkably enhanced strain-resistant performance without pre-stretching. When measured on a length scale of ~ 5 mm with a resistance of ~ 1–3 kΩ (Fig. 4a inset), the PEDOT:PSS/LiTFSI thin films coated on imprinted 3D-microstructured gel surface showed slight resistance changes up to 50% strain and only increased to two-to-three-fold higher resistance beyond 75% strain, thus surpassing the strain limit of the uniform film. Optical microscopy was then used to clarify the cracking process under strains ranging from 0 to 100% (Fig. 4b). A few microscopic cracks started to occur at 25% strain in both the uniform films and the 3D-microstructured films, but the cracks in the 3D-microstructured film were significantly shorter. This phenomenon could be attributed to the prevented crack propagation by the microscale curvature topography even at a strain of ~ 50% (Fig. S9a) [53, 54]. Furthermore, finite element simulations (Fig. S9b) indicated that the thin PEDOT:PSS/LiTFSI film coated on the convex microtextured soft gel surface allowed significantly relieved local strain of < 15% to be realized under an overall applied strain of 50%. Considering that the imprinted 3D microstructures contained random arrays of microscale bumps, those convex portions may be less strained, thus reducing the cracking probability and conserving the conductance pathways below 50% strain. Over the range from 50 to 100% strain, increasing numbers of cracks were observed on the uniform film, which greatly raised its resistance. In contrast, the crack density was lower on the 3D-microstructured film and remained unchanged beyond 50% strain. Additionally, the applied tension only induced expansion of the cracked areas, while the remaining continuous regions of the PEDOT:PSS/LiTFSI films were found to maintain a strain of ~ 30%, even under a total strain of 100%. Since the uncracked area could be much larger in the 3D-microstructured film, we speculated that if the channel was engineered to have 3D microarchitectures and a small channel area, it may be able to endure large strains of up to 100%.

Based on the knowledge that the wrinkled and 3D-imprinted microstructures enabled highly stretchable electronic conductors, we then evaluated the performances of stretchable OECTs with the two channel morphology types and wrinkled thick electrodes under strains. First, wrinkled PEDOT:PSS/LiTFSI thin channels and thick electrodes with serpentine shapes were fabricated on GEL-GLY/Na_3_Cit with uniaxial pre-strain of ~ 100% (Fig. S10a, b). As expected, along the pre-stretched axis, the OECT showed almost unchanged transfer characteristics with varying strains below 100%, under which *I*_ON_ showed variation of < 15% (Figs. 4c and S10c). Even when the device was slightly overstretched to 120%, more than 70% of *I*_D_ could be retained by harnessing the intrinsic stretchability of the PEDOT:PSS/LiTFSI. Additionally, the maximum *G*_*m*_ was also preserved even at 120% strain. Furthermore, the wrinkled electrode remained stable during cyclic stretching at 100% strain for 600 cycles, and a slight drop in current of ~ 20% was observed after 800 cycles (Fig. S10d), which was probably caused by the residual elongation of the gel substrate. We also investigated the transfer curves of OECT when it was stretched along the axis perpendicular to the pre-stretched direction. It was observed that its performance could be preserved at strains up to 40% (Fig. S10e) owing to the effect of the serpentine configuration in alleviating the local stress distribution. Nevertheless, severe fractures and loss of electronic functions were observed in this OECT after stretching at 60% strain.

In comparison, the unique 3D-imprinted channel interface with its randomly distributed curvatures could endow the OECT with identical stretchability in all directions. The advantages of this interface over the wrinkled interface would include a straightforward fabrication approach without the requirement for prestretching to reduce potential mechanical damage, guaranteed interfacial adhesion to stabilize the electron–ion transport process, and the ability to provide precise control over the 3D microstructures. We prepared OECTs with a 3D-microstructured PEDOT:PSS/LiTFSI channel (*W*/*L* = 1000/200 μm) imprinted between pre-made biaxially wrinkled electrodes to investigate the stability of the channel under biaxial stretching conditions. As shown in Fig. 4d, when the 3D-microstructured channel was stretched in parallel to the source-drain electrodes, the transfer curves nearly overlapped at *V*_G_ below 1.2 V within the strain range from 0 to 50%. Then, *I*_ON_ gradually decreased with increasing strain, but was maintained at ~ 84% of its original value under 100% strain. The value of maximum *G*_*m*_ was also retained at > 4 mS (~ 80%) and at > 3.5 mS (~ 70%) within the strain ranges of 25–50% and 75–100%, respectively, with all the *G*_*m*_ peaks appearing between 1.0 and 1.2 V (Figs. 4d(i) and e). Subsequently, the same OECT was stretched in the perpendicular direction, and more than 87% of *I*_ON_ value and > 70% of maximum *G*_*m*_ value were again preserved during stretching up to 100% strain (Fig. 4d(ii) and e). Interestingly, the *G*_*m*_ peaks were found to shift from ~ 1.0 V to more than 1.5 V with increasing strain. This change can be attributed to the extension of the distance between the channel and gate regions produced by stretching, thus reducing the effective electric field for ion motion. Therefore, the changes in device geometry that occur during stretching in the solid-state OECT may be a critical concern in terms of performance variation [22]. Furthermore, the combination of the 3D-microstructured channel and the wrinkled electrodes was also capable of withstanding 1000 stretching cycles under 80% uniaxial strain, during which no significant reductions in *I*_ON_ and *G*_*m*_ were discovered for the first 400 cycles, and retention of ~ 95% of *I*_ON_ and ~ 75% of *G*_*m*_ was ultimately achieved after 1000 cycles (Fig. 4f, g). The cracks that occur during repeated elongation and the slight residual strain in the gel electrolyte may cause *G*_*m*_ to deteriorate by impairing the channel/electrolyte interface. Despite this, the OECT performance was found to recover in terms of both *I*_ON_ (~ 99%) and *G*_*m*_ (~ 92%) after 24 h, possibly owing to the reconstructed dynamic gel networks that can heal the interface [40]. The recovered device could still be stretched up to 80% strain while exhibiting a slightly reduced *G*_*m*_ (~ 82% of the unstrained value), demonstrating the robustness of the 3D-microstructured channel interface. To the best of our knowledge, the biaxial stretchability and long-period cycling at large strains achieved by the all-polymer OECT with 3D micro-engineered interfaces exceeded the performance of most reported PEDOT:PSS-based stretchable OECTs, either with liquid or solid-state electrolytes (Table S1).

**Fig. 5:**
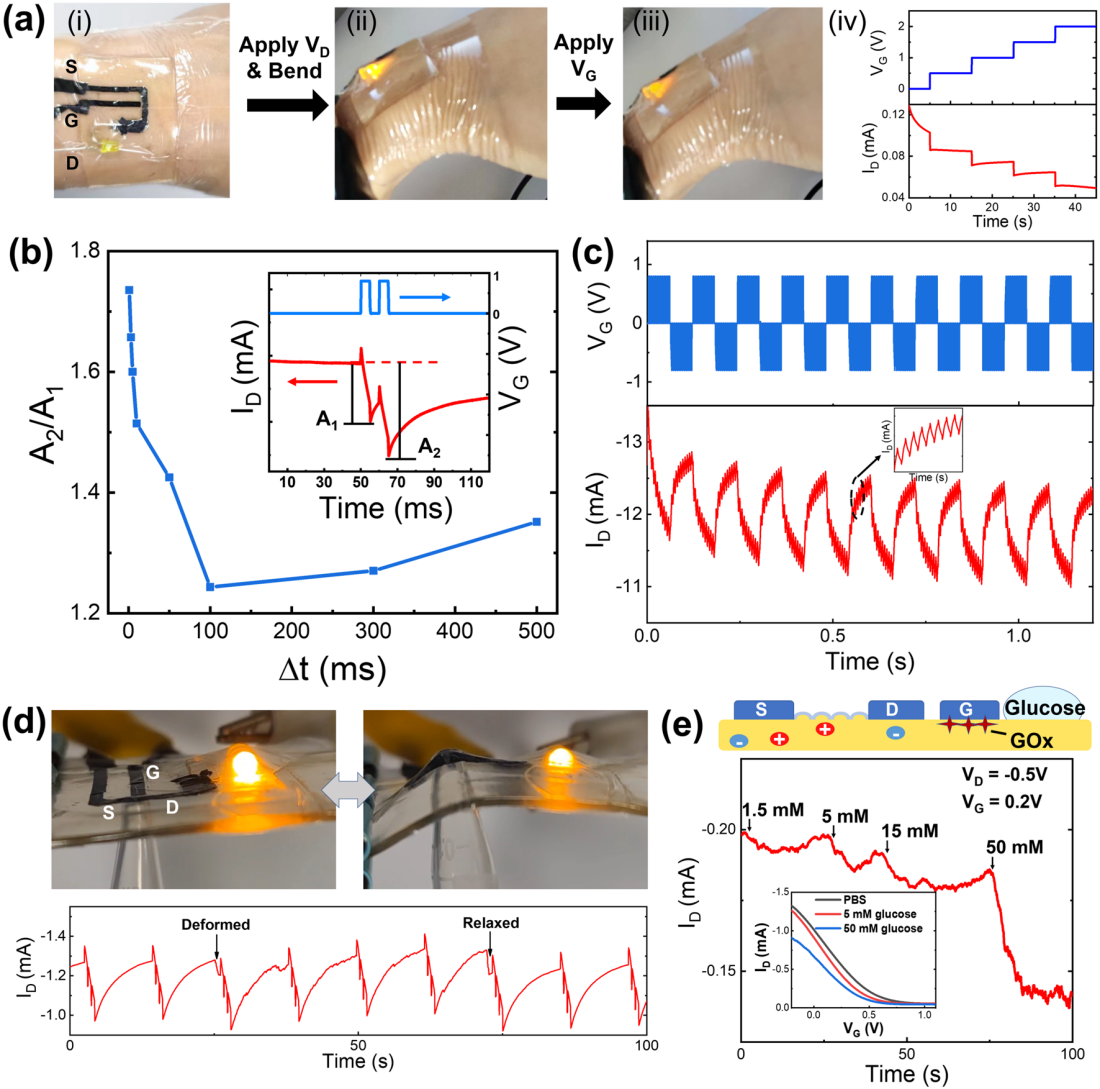
**a** Photos (i–iii) showing the on-skin circuit that used an all-polymer OECT to control the brightness of an LED in serial connection with the channel, which could be operated under wrist bending. And the corresponding *I*_D_ reduction (*V*_D_ = 2 V) along with the 0.5-V step increase of *V*_G_ (iv). **b** Paired-pulse depression (PPD) behavior of the all-polymer OECT with a spike duration (*t*_*p*_) of 5 ms and intervals (*Δt*) ranging from 500 to 1 ms. **c** Long-term potentiation (LTP) and long-term depression (LTD) behaviors with sequential 10 spikes (*t*_*p*_ = 3 ms and *Δt* = 3 ms). **d** The stability in current (top) and PPD performance (bottom, *t*_*p*_ = 500 ms and *Δt* = 100 ms) of the all-polymer OECT under omnidirectional deformation by poking the channel region. **e** Real-time plot of *I*_D_ change as dropping phosphate buffered saline (PBS) containing increasing concentration of glucose near the gate region. The inset shows the transfer curves in the presence of PBS, 5 mM and 50 mM glucose, respectively

The high elasticity of the proposed all-polymer OECTs could allow these devices to be used in a wide range of wearable applications, including skin-attachable and synapse-mimicking circuits and biosensing devices. A proof-of-concept on-skin circuit using the all-polymer OECT was demonstrated to control the brightness of a light-emitting diode (LED) bulb that was in series connection with the channel, where the entire device was attached to the wrist in a conformal fashion (Fig. 5a). Bending of the wrist did not affect the brightening of the LED bulb (Fig. 5a(ii)), thus indicating the mechanical stability of the all-polymer OECT. The increasing step change in *V*_G_ was used to effectively decrease the *I*_D_ that also flowed through the LED, so that the brightness of the LED was reduced with controlled levels (Fig. 5a(iii, iv) and Video S1). Second, synaptic plasticity behavior was also realized on the flexible devices through application of *V*_G_ using controlled spikes and intervals. The paired-pulse depression (PPD) behavior of our all-polymer OECT was examined because it is a critical function required to simulate synapses. As shown in the inset of Fig. 5b, a typical PPD curve was generated upon application of two consecutive spikes (*V*_G_ = 0.8 V). When the first spike ended, the process of deintercalation of Na^+^ from the PEDOT:PSS channel accompanied by the recovery of *I*_D_ was slower than the spike-induced drop in *I*_D_. Therefore, the next spike led to an even lower *I*_D_ drop than the previous spike. For example, the first peak value (A1) was 0.35 mA and the second peak value (A2) reached 0.56 mA with the spike duration (*t*_*p*_) of 5 ms and the interval (Δ*t*) of 5 ms. The change index (A2/A1) increased with decreasing spike intervals below 100 ms (*t*_*p*_ = 5 ms, Fig. 5b), in consistent with literatures [4, 12]. Therefore, by increasing the spike frequency to more than 10 Hz, a strong suppression effect would be induced, which indicates that a dynamic filter function similar to that of biological synapses could be realized with significant current alterations in response to high-frequency signals. Figure 5c illustrates classical long-term synaptic depression (potentiation) behavior of the gel-based OECT using Au electrodes, in which the continuous reductions and increases in *I*_D_ were analogous to the long-term behavior of synapses in response to 10 identical positive or negative spikes (*t*_*p*_ = 3 ms and Δ*t* = 3 ms). Furthermore, the operation of the LED circuit and the synapse-like behavior were tested under omnidirectional deformation conditions by poking the channel region (Fig. 5d). Once more, the current in the circuit did not show distinct variations during the poking, as indicated by the unchanged LED brightness. We then generated a string of PPD curves in this all-polymer OECT, with each having three gradually increased peaks induced by three consecutive spikes (*V*_G_ = 1.0 V, *t*_*p*_ = 500 ms and Δ*t* = 100 ms). Such PPD behavior was found to be barely disturbed when the channel was deformed and after the stress was removed, exhibiting outstanding reliability.

In addition, by using the gel networks to encapsulate bioactive molecules, we also verified the potential of the proposed all-polymer OECT for wearable biosensing applications, e.g., by modifying the gate interface with immobilized glucose oxidase (GOx) in the gelatin electrolyte. Based on the catalytic oxidation of glucose accompanied by H_2_O_2_ decomposition on the PEDOT:PSS gate, an effective *V*_G_ shift could be induced and read out based on the *I*_D_ change [31]. In detail, a drop of glucose solution was deposited near the gate, and the glucose molecules could then rapidly diffuse to the GOx-modified gate through the underlying gelatin organohydrogel to induce a change in *I*_D_. As the glucose concentration increased, *I*_D_ decreased in both the transfer curves and the real-time current response plot shown in Fig. 5e, with a detection limit of ~ 1.5 mM. Furthermore, although the gel electrolyte promoted analyte absorption, the actual analyte concentration around the gate was difficult to determine in the preliminary demonstration, showing inadequacy to provide quantitative analysis like conventional OECT-based sensing with liquid electrolyte [31]. In future studies, gate electrodes with higher catalytic activity (e.g., Pt electrodes) and gels with fast-absorbing capabilities may improve the device sensitivity and accuracy. Overall, these proof-of-concept demonstrations have paved the way towards the development of wearable and multifunctional systems using all-polymer OECTs.

### Long-Term Durability and Recycling of the All-Polymer OECTs

**Fig. 6:**
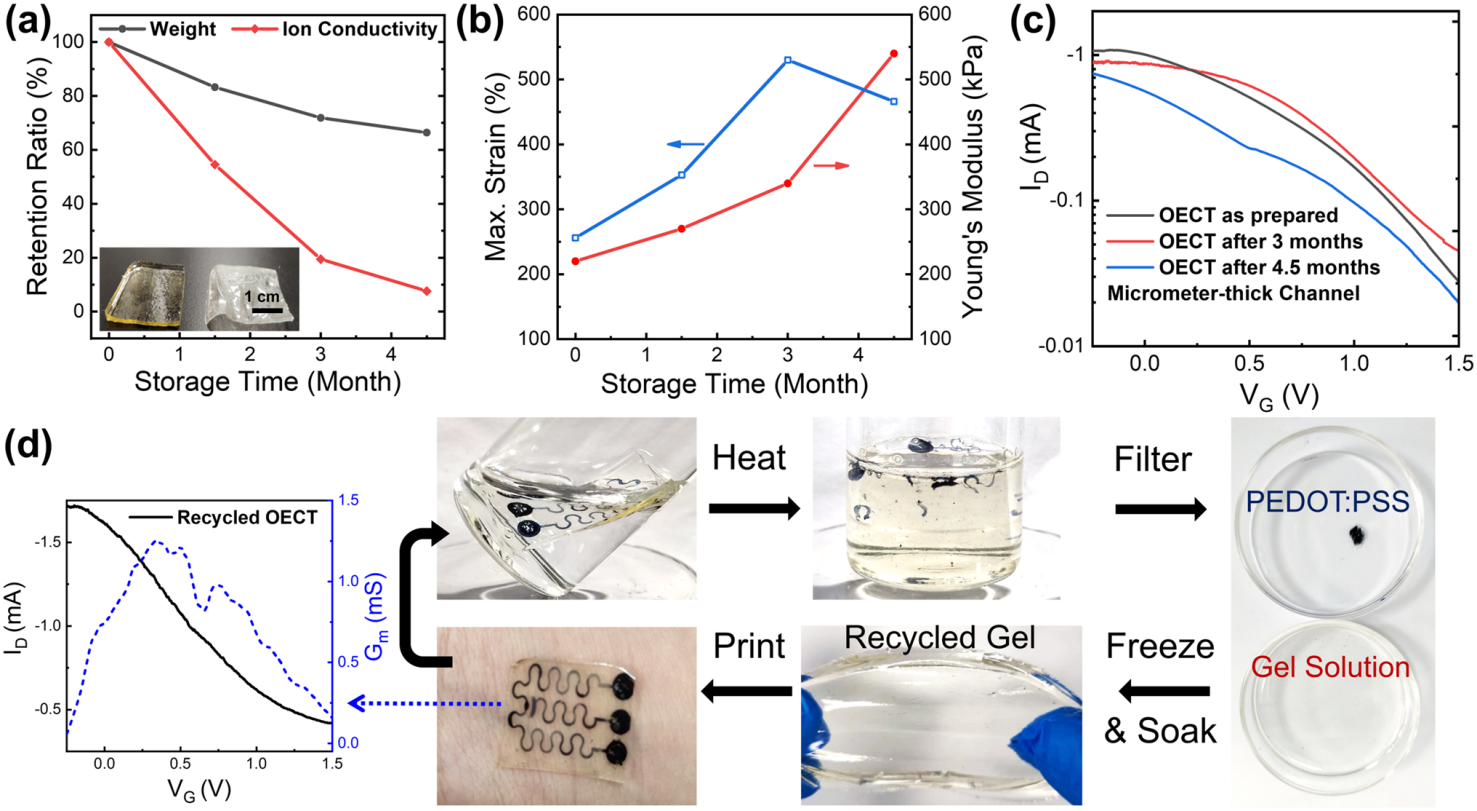
**a** Retention ratio of weight and ion conductivity for 20% GEL–60% GLY/Na_3_Cit during ambient condition storage for 4.5 months, with the inserted photo demonstrating the anti-drying nature of the glycerol-soaked gel (left) compared with gelatin hydrogel (right). **b** Plots of fracture strain and Young's modulus of the gelatin organohydrogel with the storage time. **c** Transfer curves of an all-polymer OECT measured at the initial state and after months of storage, with the channel thickness more than 1 μm. **d** Recycling of the all-polymer OECT and performance of the OECT using the recycled gel electrolyte

Finally, we explored whether or not the all-polymer OECTs would be able to adapt to various environmental conditions. First, the moisture-retaining glycerol could prevent dehydration of the gel electrolyte for up to a year [39, 40]. During ambient storage of a gelatin electrolyte soaked with 60% glycerol, the weight loss was determined to be ~ 30 wt% after 4.5 months, and this loss was accompanied by a reduction in the ion conductivity by one order of magnitude (Fig. 6a). Additionally, the mechanical stretchability and the Young’s modulus of the gel electrolyte increased with the prolonged storage, exceeding 450% strain and 500 kPa after 4.5 months, respectively, because of the elevated proportions of gelatin and glycerol in the networks (Figs. 6b and S11a). However, these aged gel electrolytes could still be used to fabricate stretchable OECTs with even lower OFF currents as a result of the reduced ion conductivity, leading to better ON/OFF ratio of up to 40 (Fig. S11b). The glycerol also allowed the organohydrogel-based device to work at low temperatures without freezing, with the *G*_*m*_ of the OECT at − 15 °C even being slightly elevated when compared with that at room temperature (Fig. S11c); this may be the result of the improved protonic transport required for channel de-doping [51]. Additionally, crosslinked PEDOT:PSS/LiTFSI films with sufficient thickness (> 1 μm) could also conserve their electrical properties for months after being pasted onto the gel electrolyte, leading to durable OECT device with > 80% of the *I*_ON_ and the maximum *G*_*m*_ values being retained after storage in the open air for more than four months (Fig. 6c). It should also be noted that the channel thickness had a significant impact on the device stability, meaning that an ultrathin channel (e.g., 300 nm thickness) could only produce a stable performance for one month. Subsequently, both *I*_ON_ and the maximum *G*_*m*_ were found to decrease gradually, with ~ 30% of their values remaining after three months (Fig. S12). This behavior might be ascribed to the water absorption and swelling of the thin PEDOT:PSS layer. This thickness-dependent lifetime of the OECT device may be beneficial for the development of transient circuits.

Although the all-polymer OECT remained stable under ambient conditions, the reversible heat-regulated sol–gel transition of gelatin could result in the device melting at higher temperatures of > 40 °C. Nevertheless, this feature also enabled on-demand device disposal and recycling under mild conditions [40, 56]. As shown in Fig. 6d, the all-polymer OECT could be degraded in hot water at temperatures above 60 °C for 30 min, which caused the gel electrolyte to become a viscous liquid, while the crosslinked PEDOT:PSS only ruptured into pieces. This enabled separation of the electrode from the electrolyte materials by simple filtering. Additionally, the dissolved gelatin in the aqueous solution could also be condensed and cooled to obtain a recycled gelatin hydrogel along with the elastic glycerol-soaked organohydrogel electrolyte. The recycled gel electrolyte could then be reused to produce an all-polymer OECT with good performance (maximum *G*_*m*_ > 1 mS). This green and gentle recycling process could be repeated for several cycles to produce devices with reproducible stretchability. Given that the destruction of the thin PEDOT:PSS layer may also be promoted by enzymatic decomposition [9], the all-polymer OECT concept also has great potential for applications in fully degradable electronics or implanted patches, in which biocompatible and biodegradable conducting polymer electrodes could be further applied [24].

## Conclusions

In summary, a general and readily available material platform was demonstrated to realize highly elastic, durable and recyclable all-polymer OECTs. These OECTs achieved tunable transconductance up to 12.7 mS, tailored 3D-micropatterned morphologies of channel/electrolyte interfaces down to sub-10-μm scale, biaxial stretchability under > 100% strain with well-preserved performance after 1000 cycles of 80% tensile strain (~ 99% *I*_ON_ and ~ 92% *G*_*m*_ recovered after 24 h), as well as more than 4-month lifetime in ambient storage. The thick microelectrodes of LiTFSI-doped PEDOT:PSS with high conductance, insensitivity to gate bias and good stretchability were transfer printed onto the adhesive surface of gelatin-based organohydrogel electrolyte, which constituted the all-polymer OECT platform to afford high transconductance that was comparable to metal-electrode-based devices, and wide applicability to both depletion-mode and enhancement-mode channel materials. Remarkably, the imprinted 3D-microstructured channel/electrolyte interfaces in combination with wrinkled electrodes were shown effective to stabilize the electronic and ionic transport and permit outstanding mechanical robustness at biaxial strains of 100% and repeated deformations for 1000 cycles. In addition, the non-drying gels containing glycerol and the LiTFSI-crosslinked insoluble PEDOT:PSS rendered stable electrode/electrolyte interface for months, while the reversible sol–gel transition of gelatin allowed on-demand disposal and recycling of the devices. Given the simple architecture and multifunctionality of the all-polymer OECT, a broad variety of applications would be envisioned in the future. The relatively lower ON/OFF ratio (< 40) and response speed (> 0.25 s) would not necessarily limit the applications in neuromorphic devices and signal amplification, because the transconductance is kept high for low-voltage signal inputs and the operation time scale is comparable [27, 57]. Hence more complicated artificial synaptic arrays and real-time biosensing could be further explored, in which enhanced stretchability and OECT functionality are achievable by utilizing other intrinsically stretchable conducting polymers [18, 23, 58] and ion gels [8], along with optimized 3D architectures [7] and functionalization at the interfaces [59]. Thus, we believe the proposed elastic all-polymer OECT platform may accelerate the advancement of organic electronic devices in research fields including human–machine interfaces, artificial intelligence, and sustainable/transient electronics.

## Supplementary Information

Below is the link to the electronic supplementary material.

Supplementary file1 (PDF 1160 KB)

Supplementary file2 (MP4 1649 KB)

## References

[1] Rivnay J, Inal S, Salleo A, Owens RM, Berggren M (2018). Organic electrochemical transistors. Nat. Rev. Mater..

[2] Ersman PA, Lassnig R, Strandberg J, Tu D, Keshmiri V (2019). All-printed large-scale integrated circuits based on organic electrochemical transistors. Nat. Commun..

[3] Nawaz A, Liu Q, Leong WL, Fairfull-Smith KE, Sonar P (2021). Organic electrochemical transistors for in vivo bioelectronics. Adv. Mater..

[4] Burgt Y, Lubberman E, Fuller EJ, Keene ST, Faria GC (2017). A non-volatile organic electrochemical device as a low-voltage artificial synapse for neuromorphic computing. Nat. Mater..

[5] Cea C, Spyropoulos GD, Jastrzebska-Perfect P, Ferrero JJ, Gelinas JN (2020). Enhancement-mode ion-based transistor as a comprehensive interface and real-time processing unit for in vivo electrophysiology. Nat. Mater..

[6] Molina-Lopez F, Gao TZ, Kraft U, Zhu C, Ohlund T (2019). Inkjet-printed stretchable and low voltage synaptic transistor array. Nat. Commun..

[7] Wang H, Wang W, Xie Z (2022). Patterning meets gels: advances in engineering functional gels at micro/nanoscales for soft devices. J. Polym. Sci..

[8] Wang D, Zhao S, Yin R, Li L, Lou Z (2021). Recent advanced applications of ion-gel in ionic-gated transistor. npj Flex Electron.

[9] Jo YJ, Kim H, Ok J, Shin YJ, Shin JH (2020). Biocompatible and biodegradable organic transistors using a solid-state electrolyte incorporated with choline-based ionic liquid and polysaccharide. Adv. Funct. Mater..

[10] Lee H, Lee S, Lee W, Yokota T, Fukuda K (2019). Ultrathin organic electrochemical transistor with nonvolatile and thin gel electrolyte for long-term electrophysiological monitoring. Adv. Funct. Mater..

[11] Melianas A, Quill TJ, LeCroy G, Tuchman Y, Loo H (2020). Temperature-resilient solid-state organic artificial synapses for neuromorphic computing. Sci. Adv..

[12] Han S, Yu S, Hu S, Chen H, Wu J (2021). A high endurance, temperature-resilient, and robust organic electrochemical transistor for neuromorphic circuits. J. Mater. Chem. C.

[13] Ho JC, Lin YC, Chen CK, Hsu LC, Chen WC (2022). Hydrogel-based sustainable and stretchable field-effect transistors. Org. Electron..

[14] Dai X, Vo R, Hsu HH, Deng P, Zhang Y (2019). Modularized field-effect transistor biosensors. Nano Lett..

[15] Chen S, Surendran A, Wu X, Leong WL (2020). Contact modulated ionic transfer doping in all-solid-state organic electrochemical transistor for ultra-high sensitive tactile perception at low operating voltage. Adv. Funct. Mater..

[16] Xie Z, Zhuge C, Zhao Y, Xiao W, Fu Y (2022). All-solid-state vertical three-terminal N-type organic synaptic devices for neuromorphic computing. Adv. Funct. Mater..

[17] Dai Y, Hu H, Wang M, Xu J, Wang S (2021). Stretchable transistors and functional circuits for human-integrated electronics. Nat. Electron..

[18] Chen J, Huang W, Zheng D, Xie Z, Zhuang X (2022). Highly stretchable organic electrochemical transistors with strain-resistant performance. Nat. Mater..

[19] Wang Y, Zhu CX, Pfattner R, Yan HP, Jin LH (2017). A highly stretchable, transparent, and conductive polymer. Sci. Adv..

[20] Li Y, Zhang S, Li X, Unnava VRN, Cicoira F (2019). Highly stretchable PEDOT:PSS organic electrochemical transistors achieved via polyethylene glycol addition. Flex. Print. Electron..

[21] Zhang S, Li Y, Tomasello G, Anthonisen M, Li X (2019). Tuning the electromechanical properties of PEDOT:PSS films for stretchable transistors and pressure sensors. Adv. Electron. Mater..

[22] Su X, Wu X, Chen S, Nedumaran AM, Stephen M (2022). A highly conducting polymer for self-healable, printable, and stretchable organic electrochemical transistor arrays and near hysteresis-free soft tactile sensors. Adv. Mater..

[23] Tan P, Wang H, Xiao F, Lu X, Shang W (2022). Solution-processable, soft, self-adhesive, and conductive polymer composites for soft electronics. Nat. Commun..

[24] Jiang Y, Zhang Z, Wang YX, Li D, Coen CT (2022). Topological supramolecular network enabled high-conductivity, stretchable organic bioelectronics. Science.

[25] Zhang SM, Hubis E, Tomasello G, Soliveri G, Kumar P (2017). Patterning of stretchable organic electrochemical transistors. Chem. Mater..

[26] Lee W, Kobayashi S, Nagase M, Jimbo Y, Saito I (2018). Nonthrombogenic, stretchable, active multielectrode array for electroanatomical mapping. Sci. Adv..

[27] Matsuhisa N, Jiang Y, Liu Z, Chen G, Wan C (2019). High-transconductance stretchable transistors achieved by controlled gold microcrack morphology. Adv. Electron. Mater..

[28] Lee Y, Oh JY, Xu W, Kim O, Kim TR (2018). Stretchable organic optoelectronic sensorimotor synapse. Sci. Adv..

[29] Kim JT, Pyo J, Rho J, Ahn JH, Je JH (2012). Three-dimensional writing of highly stretchable organic nanowires. ACS Macro Lett..

[30] Yang A, Li Y, Yang C, Fu Y, Wang N (2018). Fabric organic electrochemical transistors for biosensors. Adv. Mater..

[31] Li Y, Wang N, Yang A, Ling H, Yan F (2019). Biomimicking stretchable organic electrochemical transistor. Adv. Electron. Mater..

[32] Teng L, Ye SC, Handschuh-Wang S, Zhou XH, Gan TS (2019). Liquid metal-based transient circuits for flexible and recyclable electronics. Adv. Funct. Mater..

[33] Tao X, Liao S, Wang Y (2021). Polymer-assisted fully recyclable flexible sensors. EcoMat.

[34] Lan L, Chen J, Wang Y, Li P, Yu Y (2022). Facilely accessible porous conjugated polymers toward high-performance and flexible organic electrochemical transistors. Chem. Mater..

[35] Zhou D, Chen F, Wang J, Li T, Li B (2018). Tough protein organohydrogels. J. Mater. Chem. B.

[36] Qin Z, Dong D, Yao M, Yu Q, Sun X (2019). Freezing-tolerant supramolecular organohydrogel with high toughness, thermoplasticity, and healable and adhesive properties. ACS Appl. Mater. Interfaces.

[37] Park H, Park HW, Chung JW, Nam K, Choi S (2019). Highly stretchable, high-mobility, free-standing all-organic transistors modulated by solid-state elastomer electrolytes. Adv. Funct. Mater..

[38] Rao Z, Thukral A, Yang P, Lu Y, Shim H (2021). All-polymer based stretchable rubbery electronics and sensors. Adv. Funct. Mater..

[39] Baumgartner M, Hartmann F, Drack M, Preninger D, Wirthl D (2020). Resilient yet entirely degradable gelatin-based biogels for soft robots and electronics. Nat. Mater..

[40] Fang L, Zhang J, Wang W, Zhang Y, Chen F (2020). Stretchable, healable, and degradable soft ionic microdevices based on multifunctional soaking-toughened dual-dynamic-network organohydrogel electrolytes. ACS Appl. Mater. Interfaces.

[41] Li X, Liu Z, Zhou Z, Gao H, Liang G (2020). Effects of cationic species in salts on the electrical conductivity of doped PEDOT:PSS films. ACS Appl. Polym. Mater..

[42] Li Q, Deng M, Zhang S, Zhao D, Jiang Q (2019). Synergistic enhancement of thermoelectric and mechanical performances of ionic liquid LiTFSI modulated PEDOT flexible films. J. Mater. Chem. C.

[43] Yan J, Qin Y, Fan WT, Wu WT, Lv SW (2021). Plasticizer and catalyst co-functionalized PEDOT:PSS enables stretchable electrochemical sensing of living cells. Chem. Sci..

[44] Wang J, Li Q, Li K, Sun X, Wang Y (2022). Ultra-high electrical conductivity in filler-free polymeric hydrogels toward thermoelectrics and electromagnetic interference shielding. Adv. Mater..

[45] Rivnay J, Leleux P, Ferro M, Sessolo M, Williamson A (2015). High-performance transistors for bioelectronics through tuning of channel thickness. Sci. Adv..

[46] Wu X, Surendran A, Ko J, Filonik O, Herzig EM (2019). Ionic-liquid doping enables high transconductance, fast response time, and high ion sensitivity in organic electrochemical transistors. Adv. Mater..

[47] Wang W, Chen F, Fang L, Li Z, Xie Z (2022). Reversibly stretchable organohydrogel-based soft electronics with robust and redox-active interfaces enabled by polyphenol-incorporated double networks. ACS Appl. Mater. Interfaces.

[48] Kang S, Lee BY, Lee SH, Lee SD (2019). High resolution micro-patterning of stretchable polymer electrodes through directed wetting localization. Sci. Rep..

[49] Zhang S, Ling H, Chen Y, Cui Q, Ni J (2019). Hydrogel-enabled transfer-printing of conducting polymer films for soft organic bioelectronics. Adv. Funct. Mater..

[50] Jo YJ, Kwon KY, Khan ZU, Crispin X, Kim TI (2018). Gelatin hydrogel-based organic electrochemical transistors and their integrated logic circuits. ACS Appl. Mater. Interfaces.

[51] Nguyen-Dang T, Harrison K, Lill A, Dixon A, Lewis E (2021). Biomaterial-based solid-electrolyte organic electrochemical transistors for electronic and neuromorphic applications. Adv. Electron. Mater..

[52] Ko J, Wu X, Surendran A, Muhammad BT, Leong WL (2020). Self-healable organic electrochemical transistor with high transconductance, fast response, and long-term stability. ACS Appl. Mater. Interfaces.

[53] Fang L, Cai Z, Ding Z, Chen T, Zhang J (2019). Skin-inspired surface-microstructured tough hydrogel electrolytes for stretchable supercapacitors. ACS Appl. Mater. Interfaces.

[54] Guo R, Yu Y, Zeng J, Liu X, Zhou X (2015). Biomimicking topographic elastomeric petals (E-petals) for omnidirectional stretchable and printable electronics. Adv. Sci..

[55] Corletto A, Shapter JG (2021). High-resolution and scalable printing of highly conductive PEDOT:PSS for printable electronics. J. Mater. Chem. C.

[56] Qin Z, Sun X, Zhang H, Yu Q, Wang X (2020). A transparent, ultrastretchable and fully recyclable gelatin organohydrogel based electronic sensor with broad operating temperature. J. Mater. Chem. A.

[57] Shen H, Abtahi A, Lussem B, Boudouris BW, Mei J (2021). Device engineering in organic electrochemical transistors toward multifunctional applications. ACS Appl. Electron. Mater..

[58] Dai Y, Dai S, Li N, Li Y, Moser M (2022). Stretchable redox-active semiconducting polymers for high-performance organic electrochemical transistors. Adv. Mater..

[59] Sun C, Wang X, Auwalu MA, Cheng S, Hu W (2021). Organic thin film transistors-based biosensors. EcoMat.

