# Supplementary material for: High-Transconductance, Highly Elastic, Durable and Recyclable All-Polymer Electrochemical Transistors with 3D Micro-Engineered Interfaces
Source: Nanomicro Lett. 2022 Sep 12;14:184. doi: 10.1007/s40820-022-00930-5 (PMC9468203; doi:10.1007/s40820-022-00930-5)
Supplement: Supplementary file 1 — Supplementary file1 (PDF 1160 KB) [file 40820_2022_930_MOESM1_ESM.pdf]

Supporting Information for

High-Transconductance, Highly Elastic, Durable and Recyclable All-Polymer Electrochemical Transistors with 3D Micro-Engineered Interfaces

Wenjin Wang[1, #], Zhaoxian Li[1, #], Mancheng Li[1], Lvye Fang[1], Fubin Chen[1], Songjia Han[2], Liuyuan Lan[1], Junxin Chen[1], Qize Chen[1], Hongshang Wang[1], Chuan Liu[2], Yabin Yang[1], Wan Yue[1], Zhuang Xie[1, *]

[1] School of Materials Science and Engineering, Guangzhou Key Laboratory of Flexible Electronic Materials and Wearable Devices, and Key Laboratory for Polymeric Composite and Functional Materials of Ministry of Education, Sun Yat-sen University, Guangzhou, 510275, P. R. China

[2] State Key Laboratory of Optoelectronic Materials and Technologies and Guangdong Province Key Laboratory of Display Material and Technology, School of Electronics and Information Technology, Sun Yat-Sen University, Guangzhou 510275, P. R. China

[#] Wenjin Wang and Zhaoxian Li contributed equally to this work.

*Corresponding author. E-mail: xiezhuang@mail.sysu.edu.cn (Zhuang Xie)

Supplementary Figures and Table

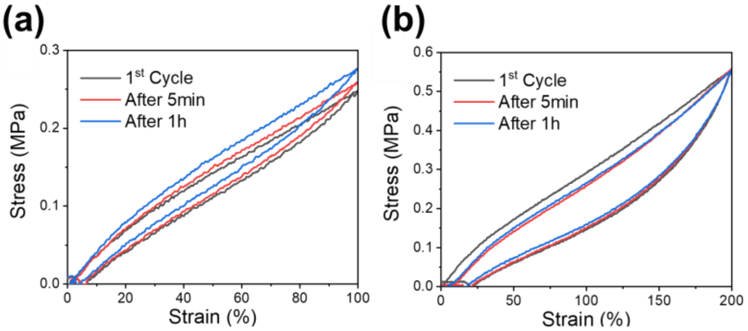

Fig. S1 Cyclic loading–unloading curves at 100% (**a**) and 200% (**b**) strains for 25% GEL-60% GLY/Na_3Cit, with diminished residual strain and recovery of dissipated energy observed after 5 min

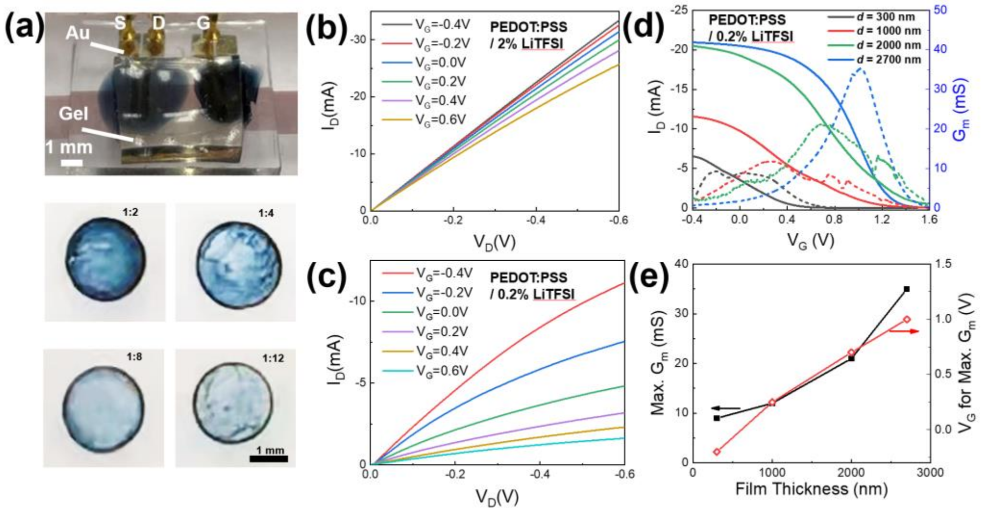

Fig. S2 **a** Photos of the OECT measurement on Au electrodes (*W/L* = 5000/200 μm) and PEDOT:PSS/LiTFSI with varied thickness through controlling the dilution ratio. **b-c** Typical output curves of PEDOT:PSS doped with 2 wt% LiTFSI (**b**) and 0.2 wt% LiTFSI (**c**) with

similar thickness (1:8 dilution). **d** Control of the transfer and G_m characteristics of PEDOT:PSS/0.2% LiTFSI channels by adjusting the thickness as measured by atomic force microscopy (AFM). **e** The corresponding plots of the max. G_m and the V_G for the max. G_m with channel thickness

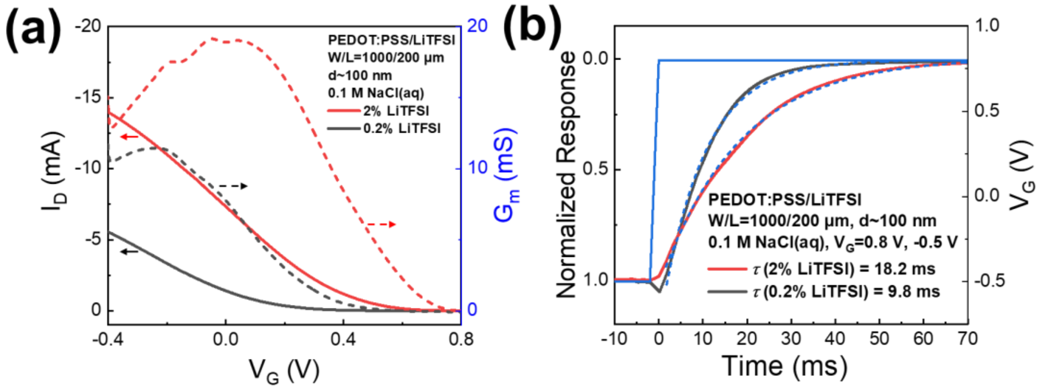

Fig. S3 a Transfer and G_m curves and **b** normalized temporal responses of PEDOT:PSS channels (~100-nm thick) with 0.2 wt% LiTFSI and 2 wt% LiTFSI, respectively, which were drop cast on Au electrodes (W/L = 1000/200 μm) and gated with Ag/AgCl through 0.1 M NaCl aqueous solution. The blue curve in (**b**) shows the applied V_G. The normalized transconductances were calculated as ~220 and ~380 S cm^{-1} for channels with 0.2 wt% LiTFSI and 2 wt% LiTFSI, respectively

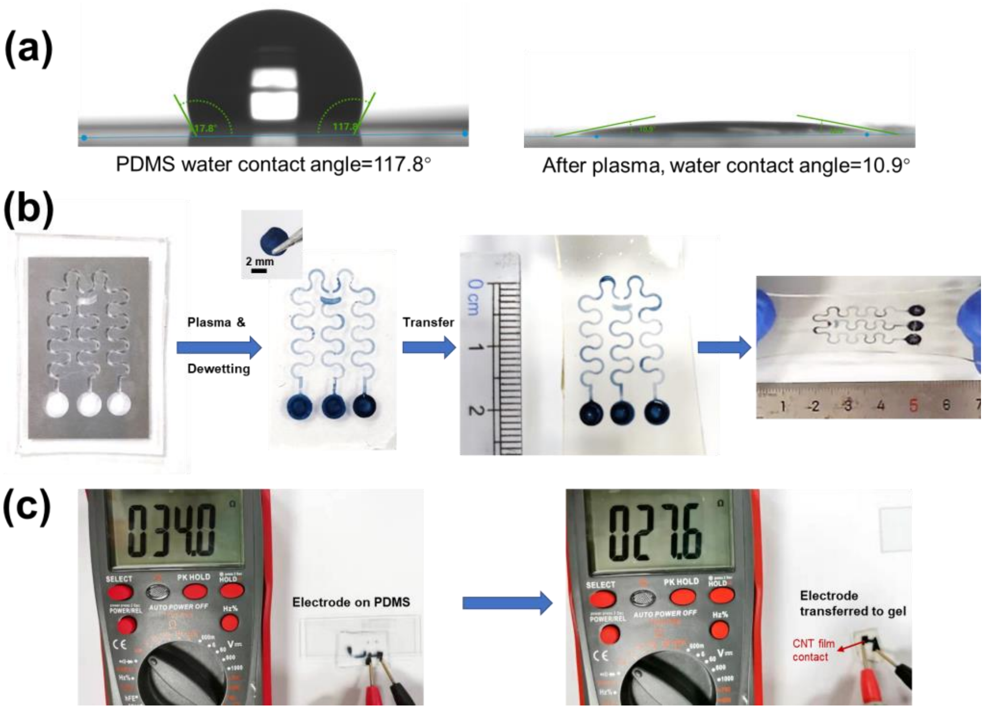

Fig. S4 a Contact angle measurements of hydrophobic PDMS substrate before and after oxygen plasma treatment. **b** Fabrication process of patterned hydrophilic and hydrophobic PDMS surface using oxygen plasma under a stainless steel mask to form the PEDOT:PSS/LiTFSI patterns on PDMS by dewetting, followed by transfer printing to obtain stretchable microelectrodes on the gel substrate. **c** Resistance values of the PEDOT:PSS/LiTFSI electrode before (left) and after (right) transfer onto GEL-GLY/Na_3Cit. The soft carbon nanotube (CNT) thin films were used for contact pads

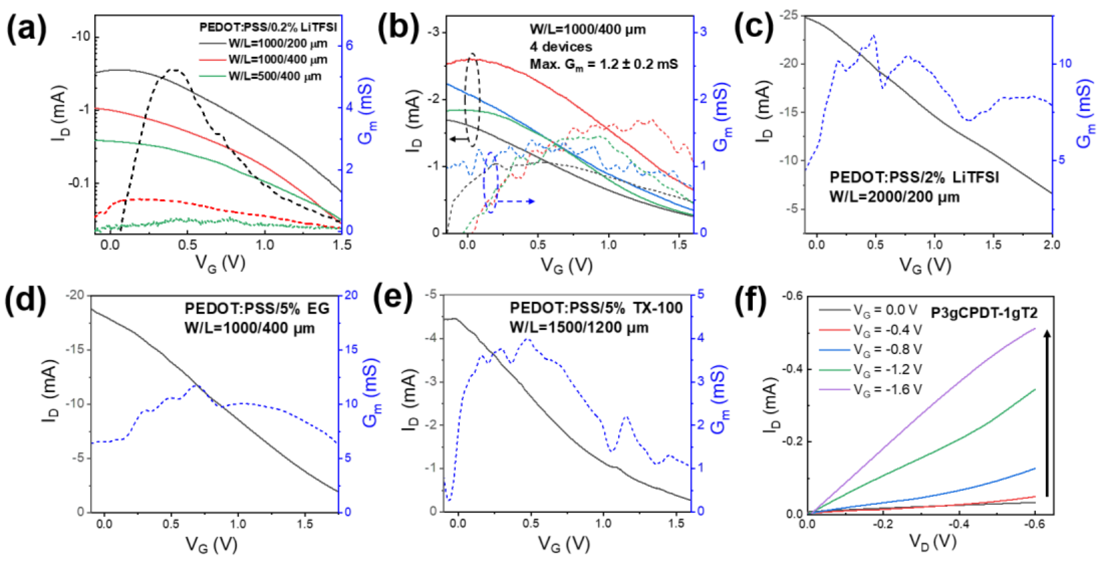

Fig. S5 a Transfer and G_m curves of all-polymer OECTs with controlled W/L ratios. The channels were PEDOT:PSS/0.2% LiTFSI with *d*<1 μm. **b** Transfer and G_m curves of 4 devices with the constant electrode design of *W/L* = 1000/400 μm to show the performance variation of the all-polymer OECTs from one batch fabrication. (**c-e**) Transfer and G_m curves of the all-polymer OECTs consisting of PEDOT:PSS/2% LiTFSI electrodes and thin channels of PEDOT:PSS/2% LiTFSI (**c**), PEDOT:PSS/5% ethylene glycol (EG, **d**) and PEDOT:PSS/5% TritonX-100 (TX-100, **e**). **f** Output characteristics of the enhancement-mode all-polymer OECT using P3gCPDT-1gT2 as the channel

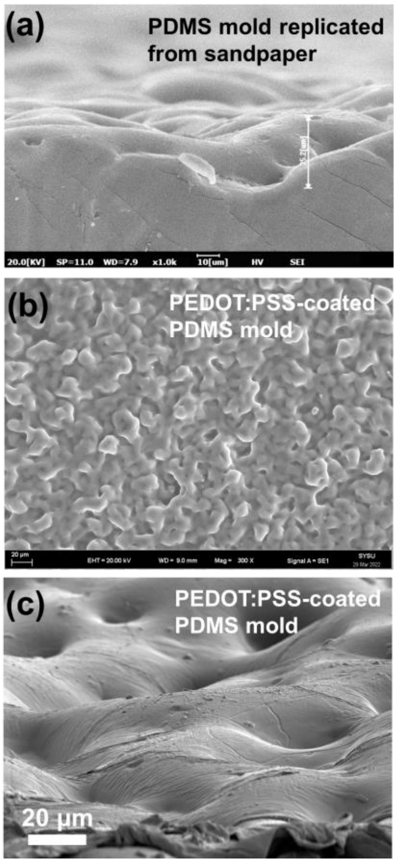

Fig. S6 a Cross-sectional scanning electron microscopy (SEM) image of PDMS mold replicated from a sandpaper (400 mesh). **b-c** SEM top (**b**) and side (**c**) view images of the PDMS mold after spin coating PEDOT:PSS/LiTFSI thin film

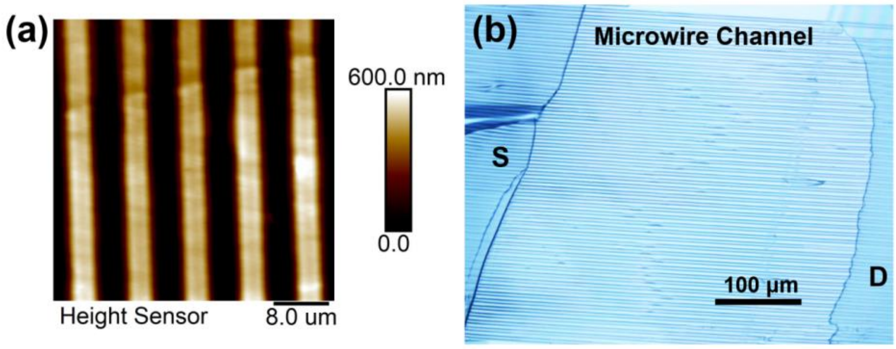

Fig. S7 a Typical AFM topography image of the printed PEDOT:PSS/LiTFSI microwire arrays on the gel electrolyte. **b** Optical microscope image of the microwire channel by printing two polymer electrodes to contact with the microwire arrays

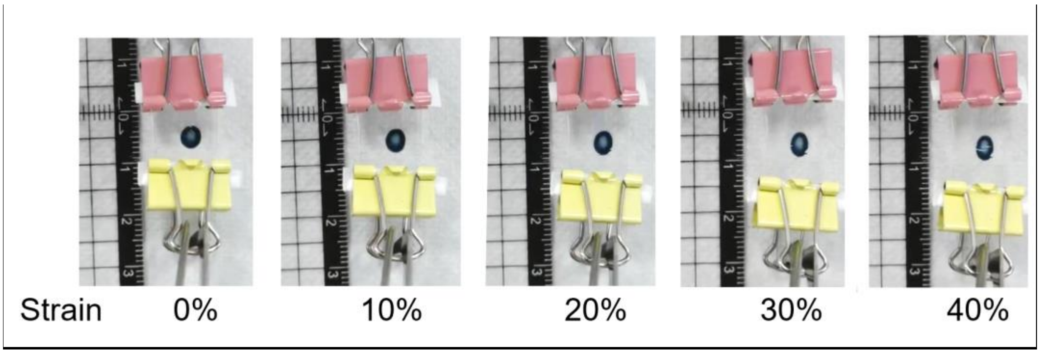

Fig. S8 Photos of PEDOT:PSS/2% LiTFSI film attached on the gel electrolyte under strains from 0-40%, showing the macroscopic cracks appearing at ~40% strain

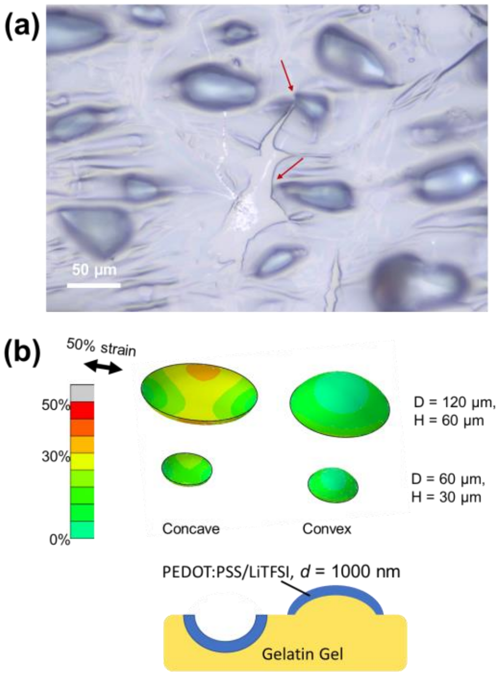

Fig. S9 a Magnified optical microscope image of the microstructured PEDOT:PSS/LiTFSI thin film on the gel substrate under ~50% strain, with the arrows indicating the prevented crack propagation at the bump regions. **b** Finite element simulations of strain distribution on PEDOT:PSS/LiTFSI thin layer coated on concave and convex microstructures of gelatin-based gel electrolyte, with an overall strain of 50%. It indicates that the convex structure and the smaller diameter may decrease the strain on the PEDOT:PSS/LiTFSI

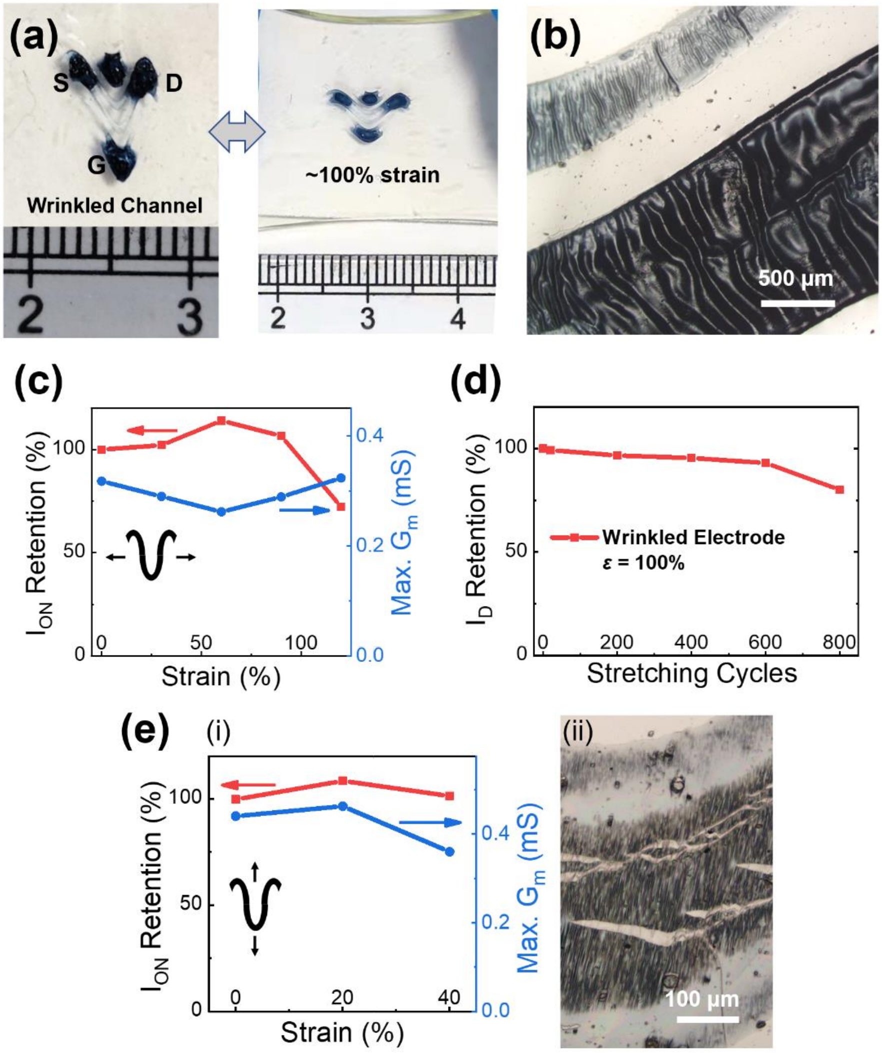

Fig. S10 a Photographs of the all-polymer OECT with wrinkled channel and electrodes (100% prestrain) under relaxed and stretched states. **b** Optical microscope image of the wrinkled channel and electrode. **c** I_{ON} retention and the maximum G_m of the stretchable all-polymer OECT during stretching from 0 to 100% strain parallel to the prestretched direction. **d** I_D retention of the wrinkled electrode under repeated stretching for 800 cycles at 100% strain. **e** OECT performance under strains of 0-40% in the perpendicular direction (**i**). The corresponding optical microscope image (**ii**) shows the channel cracking after perpendicular stretching

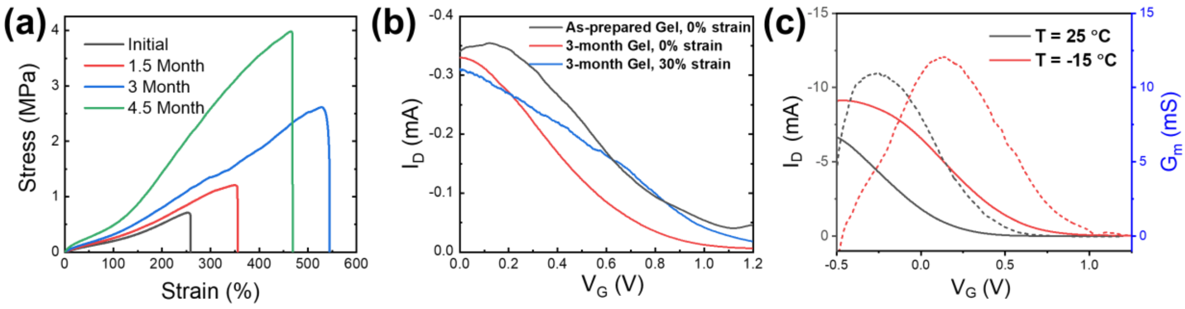

Fig. S11 a Typical tensile stress–strain curves of 20% GEL-60% GLY/Na_3Cit under ambient storage for up to 4.5 months. **b** Transfer curves of OECT prepared with fresh and aged gel electrolytes. **c** Transfer curves of gel-based OECT under varied temperature conditions

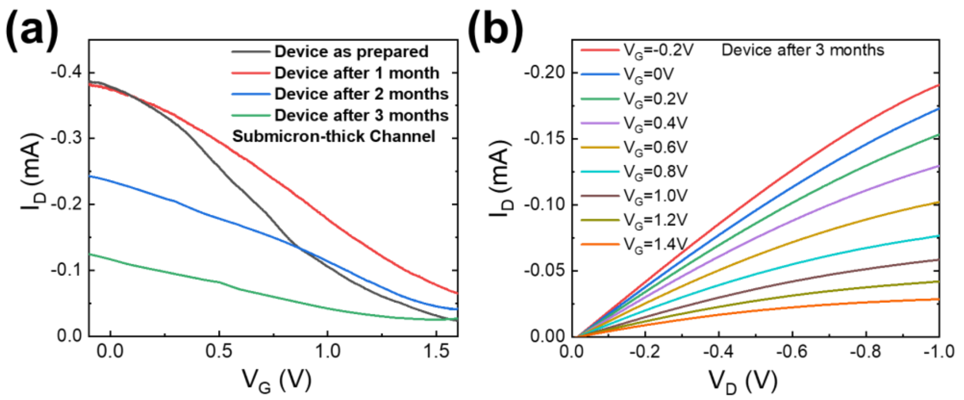

Fig. S12 a Transfer curves of an all-polymer OECT with submicron-thick channel during the storage for 3 months. **b** Output curves of the OECT after 3 months

Table S1 Comparison among stretchable OECTs using PEDOT:PSS channels

Materials	Device Dimensions	G_m	Stretch-ability	Long-term Operation	Refs.
PEDOT:PSS/glycerol/Capstone FS-30 Electrodes: wrinkled Au, carbon gate Electrolyte: PAM hydrogel	W/L=2000/ 8000 µm d=400 nm	1.1 mS Normalized: 110 S·cm^{-1}	50 %	1000 cycles at 30 % strain	[S1]
PEDOT:PSS/EG/GOPS Electrodes: Au Electrolyte: aqueous solution	W/L=50/50 µm d<200 nm	1 mS Normalized: >50 S·cm^{-1}	15 %	1000 cycles at 15 % strain	[S2]
PEDOT:PSS/EG/GOPS Electrodes: wrinkled Au Electrolyte: PAM/PVA/glycerol organohydrogel	W/L=50/50 µm d<200 nm	1.62 mS Normalized: >80 S·cm^{-1}	30%	1000 cycles at 20% strain, storage: 8 days	[S3]
PEDOT:PSS/EG/GOPS Electrodes: microcracked Au Electrolyte: aqueous solution	W/L=630/ 130 µm d<200 nm	0.54 mS Normalized: >6 S·cm^{-1}	100%	1000 cycles at 50 % strain	[S4]
Wavy PEDOT:PSS/glycerol/Zonyl fluoro-surfactant Electrodes: wavy Au/PEDOT:PSS Electrolyte: aqueous solution	NA	~1 mS	biaxial 30%	NA	[S5]
PEDOT:PSS/glycerol/Capstone FS-30 Electrodes: Au, carbon gate Electrolyte: aqueous solution	W/L=2000/ 8000 µm d=50 nm	0.2 mS Normalized: 160 S·cm^{-1}	30 %	60 cycles at 30 % strain	[S6]
PEDOT:PSS/glycerol/Capstone FS-30/PEG 400 Electrodes: Au, carbon gate Electrolyte: aqueous solution	W/L=2000/ 8000 µm d=300 nm	0.1 mS Normalized: 13 S·cm^{-1}	45 %	100 cycles at 45 % strain	[S7]

PEDOT:PSS/PEG/divinyl sulfone Electrodes: AuNPs-AgNWs Electrolyte: ionic liquid	W/L=2000/ 200 μm d=440 nm	27.43 mS Normalized: 62.3 $S \cdot cm^{-1}$	biaxial 30%	NA	[S8]
PEDOT:PSS/PAMPS/ionic liquid Electrodes: AgNWs, SWCNT gate Electrolyte: P(VDF-HFP) ionogel	W/L=1000/ 250 μm d=200 nm	12.95 mS Normalized: 162 $S \cdot cm^{-1}$	biaxial 100%	NA	[S9]
Microstructured PEDOT:PSS/LiTFSI Electrodes: wrinkled PEDOT:PSS/LiTFSI Electrolyte: gelatin-glycerol/Na_3Cit organohydrogel	W/L=2000/ 200 μm d=500 nm	12.7 mS Normalized: 25.4 $S \cdot cm^{-1}$	biaxial 100%, uniaxial 120%	1000 cycles at 80 % strain, storage: >4 months	This Work

Supplementary References

[S1]S.M. Zhang, E. Hubis, G. Tomasello, G. Soliveri, P. Kumar et al., Patterning of stretchable organic electrochemical transistors. Chem. Mater. **29**(7), 3126-3132 (2017). https://doi.org/10.1021/acs.chemmater.7b00181

[S2]W. Lee, S. Kobayashi, M. Nagase, Y. Jimbo, I. Saito et al., Nonthrombogenic, stretchable, active multielectrode array for electroanatomical mapping. Sci. Adv. **4**(10), eaau2426 (2018). https://doi.org/10.1126/sciadv.aau2426

[S3]H. Lee, S. Lee, W. Lee, T. Yokota, K. Fukuda et al., Ultrathin organic electrochemical transistor with nonvolatile and thin gel electrolyte for long-term electrophysiological monitoring. Adv. Funct. Mater. **29**(48), 1906982 (2019). https://doi.org/10.1002/adfm.201906982

[S4]N. Matsuhisa, Y. Jiang, Z. Liu, G. Chen, C. Wan et al., High-transconductance stretchable transistors achieved by controlled gold microcrack morphology. Adv. Electron. Mater. **5**(8), 1900347 (2019). https://doi.org/10.1002/aelm.201900347

[S5]Y. Li, N. Wang, A. Yang, H. Ling, F. Yan, Biomimicking stretchable organic electrochemical transistor. Adv. Electron. Mater. **5**(10), 1900566 (2019). https://doi.org/10.1002/aelm.201900566

[S6]S. Zhang, Y. Li, G. Tomasello, M. Anthonisen, X. Li et al., Tuning the electromechanical properties of PEDOT:PSS films for stretchable transistors and pressure sensors. Adv. Electron. Mater. **5**(6), 1900191 (2019). https://doi.org/10.1002/aelm.201900191

[S7]Y. Li, S. Zhang, X. Li, V.R.N. Unnava, F. Cicoira, Highly stretchable PEDOT:PSS organic electrochemical transistors achieved via polyethylene glycol addition. Flex. Print. Electron. **4**, 044004 (2019). https://doi.org/10.1088/2058-8585/ab5202

[S8]S. Bontapalle, M. Na, H. Park, K. Sim, Fully soft organic electrochemical transistor enabling direct skin-mountable electrophysiological signal amplification. Chem. Commun. **58**, 1298 (2022). https://doi.org/10.1039/D1CC04884H

[S9]X. Su, X. Wu, S. Chen, A.M. Nedumaran, M. Stephen et al., A highly conducting polymer for self-healable, printable, and stretchable organic electrochemical transistor arrays and near hysteresis-free soft tactile sensors. Adv. Mater. **34**(19), 2200682 (2022). https://doi.org/10.1002/adma.202200682
